# A survey of the clinical usage of intra-articular therapeutics in dogs by veterinary practitioners with a focus on non-steroidal therapies

**DOI:** 10.3389/fvets.2026.1761681

**Published:** 2026-02-11

**Authors:** Camila P. Sepulveda, Kayla M. Corriveau, Lindsey H. Boone, Erik H. Hofmeister, Margaret R. Kane, Kara M. Lascola, Anne A. Wooldridge

**Affiliations:** 1 Department of Clinical Sciences, College of Veterinary Medicine, Auburn University, Auburn, AL, United States; 2 Chesapeake Veterinary Surgical Specialists, Ethos Veterinary Health, Annapolis, MD, United States

**Keywords:** canine, intra-articular, non-steroidal therapeutics, orthobiologics, osteoarthritis, radionuclide, survey, viscosupplements

## Abstract

Osteoarthritis (OA) is a degenerative condition of the joint, whose progressive characteristics can lead to significant functional impairment and pain in the canine patient. Interest has increased regarding the use of intra-articular (IA) therapeutics as part of the multimodal management of canine OA. This study aimed to present the clinical experience of surgery and rehabilitation practitioners using these therapeutics to better understand their clinical usage, perceived outcomes, and clinical reasoning for product usage. A survey inquiring about IA injections with a focus on non-steroidal intra-articular therapeutics (NSIATs), including platelet-rich plasma (PRP), autologous conditioned serum (ACS), autologous protein solution (APS), cellular therapy, viscosupplements, and radionuclide, was distributed to canine practitioners. A total of 174 surveys were included in the results. Intra-articular injections were performed by 164 participants. Among these, 144 practitioners used NSIATs. The most common joint injected with steroidal and/or NSIATs was the elbow. The top reason for participants’ decision as to which NSIATs they preferentially used was scientific data and articles published regarding the product’s safety and efficacy. The most used NSIATs were PRP and viscosupplements, followed by cellular therapy, radionuclides, ACS, and APS. Practitioners reported that the most common reason to use NSIATs, except for APS, was chronic articular pathology needing ‘maintenance’ or routine injections. Pertaining to the top two most commonly used products, most participants did not combine PRP with other IA therapeutics. In the case of viscosupplements, 40% of the practitioners combined them with corticosteroids. According to the participants’ subjective assessment, most of the positive responders presented substantial or some clinical improvement after PRP or viscosupplements administration. As the first survey on this topic of small animal studies, these results provide valuable insights into the use of NSIATs in canines.

## Introduction

Osteoarthritis (OA) is a degenerative condition in canine patients characterized by progressive functional impairment and pain, presenting one of the foremost challenges in veterinary medicine as a result of the financial and emotional impact imposed on dog owners (1, 2).

Intra-articular therapy is a targeted treatment option within the multimodal management of canine OA, offering musculoskeletal benefits particularly for patients requiring long-term oral medical therapy or those unable to tolerate oral medication (3). Over the past decade, interest has increased regarding the use of non-steroidal intra-articular therapeutics (NSIATs), including blood-derived products, stem cells, and synthetic products. Research has explored various NSIATs, such as platelet-rich plasma (PRP), autologous protein solution (APS), autologous conditioned serum (ACS), cellular therapeutics, viscosupplements, radionuclide isotopes (tin-117m/SynovetinOA®), and others (4–14).

A survey of canine practitioners asking about clinical usage, injection frequency, perceived outcomes, and clinical reasoning for intra-articular (IA) product usage has not been performed. Given the increasing use of NSIATs, a better understanding of the identity, frequency, and clinical reasoning for preferences of NSIATs used by practitioners is critical. This survey was not a hypothesis-driven study. Specific hypotheses about responses could not be generated as no prior equivalent studies were available. The objective of this study was to describe current clinical use and perceived patient response to IA therapies in dogs, with a focus on blood-derived products (PRP, ACS, APS), cellular therapeutics (tissue particles, cellular concentrates, cultured cellular produces), viscosupplements (hyaluronic acid, polyacrylamide hydrogels, collagen-elastin), and radionuclide (tin-117m/SynovetinOA®). Information regarding the complications, treatment protocols, and perceived efficacy of NSIATs by canine practitioners was collected. Information gained from this study will help guide the direction of future research in this field.

## Materials and methods

This survey originated from a similar project performed among equine practitioners (15). For the canine survey, an electronic questionnaire (Qualtrics XM software, Provo, Utah, USA) was designed for surgery and rehabilitation practitioners inquiring about IA injections and the use of 6 different canine NSIATs, including PRP, ACS, APS, cellular therapeutics, viscosupplements, and radionuclide.

The survey collected demographic information about each practitioner’s practice and experience, geographic location, frequency of IA injection, and overall usage of NSIATs. There were specific questions about each NSIAT, including clinical usage, rank and justification of product preference, treatment protocol used, subjective assessment of clinical efficacy, and frequency of an observed inflammatory response (joint flare) after IA administration. For the subjective assessment of clinical efficacy, practitioners were first asked to indicate how many cases seemed to respond (i.e., in how many cases an improvement was observed) after NSIAT injection, by selecting one of the following options: few, some, a lot, or all of them. Regarding those positive responders, participants were then asked to indicate how much clinical improvement they tend to see, by selecting one of the following options: a little, some, substantial, or total resolution. If participants mentioned that they were not using NSIATs, they were asked to select the reasons for not using them in their practice.

The questionnaire contained a combination of short-answer, multiple-choice, and rank questions. It included a maximum of 71 questions, not all of which were required to be answered if the veterinarian did not use one of the products. Within the survey, brief descriptions of each NSIAT were provided before specific questions (Table 1). If the practitioner did not use a product, product questions were not considered in the future analysis. The survey was reviewed and tested by three subjects who did not include the investigators. Based on the internal test trials, the survey took approximately 15–20 min to complete all sections.

**Table 1:** Description of each NSIAT provided to survey participants before each product-specific question.

NSIATs	NSIATs description
Platelet-rich plasma (PRP)	Platelet-rich plasma (PRP) is a product obtained from the dog’s own blood. The blood is filtered or centrifuged to obtain plasma with an increased number of platelets rich in growth factors. A specific method of processing PRP is known as autologous conditioned plasma (ACP).
Autologous conditioned serum (ACS)	Autologous conditioned serum, also known as IRAP, is obtained from the dogs’ blood following collection into specialized syringes (containing treated glass beads) and whole blood incubation. The serum is then collected and administered, or aliquots are frozen for subsequent injection. Autologous conditioned serum is enriched in growth factors and anti-inflammatory cytokines like the interleukin-1 receptor antagonist (IL-1Ra).
Autologous protein solution (APS)	Autologous protein solution (i.e., Pro-Stride™ APS; nSTRIDE*®* APS) is an autologous product obtained from the dog’s blood. The blood is first processed using a separator device and centrifugation to obtain plasma with concentrated platelets. The plasma is then harvested and processed in a concentration device that allows exposure of the cellular components of the plasma to polyacrylamide beads enhancing their production of anti-inflammatory proteins during a second centrifugation cycle.
Cellular therapeutics	Cellular therapeutics would include the following products: Cells (stem/stromal and/or progenitor) contained within tissue particles. These products are typically shipped directly from the company. Progenitor and stem/stromal cell concentrates. These products are obtained after harvesting tissue (adipose or bone marrow) and concentration of the cells from the tissue via centrifugation with or without prior tissue digestion (i.e., adipose-derived stromal vascular fraction or bone marrow aspirate concentrate). Cultured cellular therapy. These products are obtained after harvesting tissue (adipose, bone marrow, blood, etc.) and sending the tissues to a commercial laboratory for culture. The cultured cells would then be shipped back to the practitioner for injection at least 2 weeks or more after the tissue harvest.
Viscosupplements	Hyaluronan, also referred to as hyaluronic acid (HA), is a naturally occurring component of cartilage and joint fluid that provides lubrication. Intra-articular injection of synthetic HA is believed to be beneficial in decreasing inflammation, repairing cartilage and decreasing symptoms of osteoarthritis (OA). Polyacrylamide hydrogel is a synthetic viscosupplement product injected intra-articularly. It is incorporated into the synovial lining and provides enhanced viscoelasticity to the synovial fluid. Collagen-elastin products are injected with the aim of providing a scaffold for intra-articular space. Other viscosupplements for intra-articular injection include glucosamine and chondroitin.
Radionuclide	Radiosynoviorthesis (RSO) is a technique used to restore the joint fluid by intra-articular injection of radioactive agents. Tin-117m colloid in the product Synovetin OA® is the most novel RSO device used to treat synovial inflammation, improve mobility and mitigate osteoarthritis in dogs.

Modified from Velloso Alvarez et al. (15).

The project was reviewed and approved by the Institutional Review Board for the Protection of Human Subjects in Research (22-462 EX 2210). The survey was distributed worldwide to an estimated 2,500 professionals between March 2024 and June 2024. The surveyed population included canine veterinary practitioners who were small and large animal Diplomates of the American College of Veterinary Surgeons (ACVS), small and large animal Diplomates of the American College of Veterinary Sports Medicine and Rehabilitation (ACVSMR), small and large animal Diplomates of the European College of Veterinary Surgeons (ECVS), small and large animal Diplomates of the European College of Veterinary Sports Medicine and Rehabilitation (ECVSMR) and/or licensed veterinarian certified as a Certified Canine Rehabilitation Therapist (CCRT), Certified Canine Rehabilitation Veterinarian (CCRV), Certified Companion Animal Rehabilitation Therapist (CCAT), Certified Canine Rehabilitation Practitioner (CCRP) and/or any other certification in animal rehabilitation not mentioned above.

Canine practitioners were contacted by email addresses obtained through university websites and veterinary practice websites, as well as ACVS, ACVSMR, ECVSMR, CCRP, CCRT, and CCAT official websites. No official CCRV email list was available on the web. The following associations, brands and canine practitioner groups distributed the questionnaire link through their Facebook pages and/or newsletters: International Veterinary Academy of Pain Management, International Association of Veterinary Rehabilitation Therapists, Canine Arthritis Management, Canine Rehabilitation Institute, Veterinary Academy of Higher Learning, The Animal Rehab Group, and ArthramidVet®. The questionnaire link was also posted in social media groups (Facebook groups: ACVS, OA & Pain Management for Veterinary Professionals, MyLameDogsVet and Animal Rehab, Physio, and Fitness Products and Resources). Lastly, a flyer and an information letter with the questionnaire link and QR code were sent by mail to 1,598 small and large animal Diplomates of the ACVS and 84 CCRT.

Any data obtained in connection with this study remained anonymous. Each participant was given a unique identifier based on email and IP address to avoid duplication of answers. A copy of the survey is provided (Supplementary file 1). After the survey closed, survey responses were downloaded into comma-separated values (CSV) format for data analysis. For all rank-style questions, only the minimum required response was included in the results. Responses were screened to exclude those who provided inconsistent answers.

### Statistical analysis

Survey data were summarized and reported using absolute values, percentages, and/or rankings. Fisher’s exact test analyses (GraphPad Prism version 10.3.1 [509] for Windows, GraphPad Software, LLC) were conducted to evaluate the influence of canine orthopedic percentile (the proportion of orthopedic cases relative to the overall canine caseload), major disciplinary focus, workplace setting, single and multiple specialty board/ certification status, and number of dogs injected intra-articularly in the last 3 months. The same test analysis was used to evaluate the influence of canine orthopedic percentile, major disciplinary focus, workplace setting, and practitioner’s geographic location with the use of PRP. The influence of canine orthopedic percentile, practitioner’s geographic location, and workplace setting with the use of viscosupplements was also evaluated using Fisher’s exact test analyses. Multiple pair Fisher’s exact test analyses and a Bonferroni correction were conducted to evaluate the influence of practitioner geographic location inside the USA and to evaluate disciplinary focus with the use of viscosupplements. Differences in perceived outcomes with the use of PRP, cellular therapy, and viscosupplements were evaluated using Fisher’s exact test analyses. Chi-Square analyses (GraphPad Prism version 10.3.1 [509] for Windows, GraphPad Software, LLC) were conducted to evaluate the practitioner’s geographic location (USA vs. non-USA residents). Unpaired t-test analyses (GraphPad Prism version 10.3.1 [509] for Windows, GraphPad Software, LLC) were conducted to evaluate the year of acquisition of the veterinary license with the use of NSIATs. Mann–Whitney test analyses (GraphPad Prism version 10.3.1 [509] for Windows, GraphPad Software, LLC) were conducted to evaluate the year that practitioners obtained their specialty board and certification status and to evaluate the influence of the year of licensure on the use of PRP and viscosupplements. Differences were considered significant when *p* < 0.05, except in the Bonferroni correction, where differences were considered significant when *p* < 0.005.

## Results

A total of 257 practitioners participated. Of these, 179 surveys were completed, and 78 were partially completed. Ultimately, 174/257 [67.7%] completed surveys were included in the results. Five completed surveys were excluded from the results for the following reasons: survey practitioners experienced issues with multiple rank questions, rendering the data unusable for the final analysis (*n* = 3); the responses given pertained to an intravenous administration and not an IA administration of the NSIAT (*n* = 1); the participant gave inconsistent responses about IA therapy and the use of NSIATs (*n* = 1).

Inconsistent answers were found in the following question: “Please rank the intra-articular products that you use most commonly in your practice to treat canine joint-related pathology from most used (1) to least used (2–8). Leave the products that you do not use blank.” In some surveys, the practitioner’s selection of an IA product(s) in this question did not match with the selection of the same IA product-specific questions asked later in the survey. For this reason, this question was excluded from the analysis of all surveys.

### Demographics

Of their total patient caseload, most practitioners indicated that their canine caseload was between 76 and 100% (127/174; [73.0%]). Forty-one of 174 [23.6%] reported a caseload between 51 and 75% canine, 4/174 [2.3%] mentioned a caseload between 26 and 50% canine and 2/174 [1.1%] reported a caseload between 0 and 25% canine. From this canine caseload, 67/174 [38.5%] of the practitioners mentioned having a canine orthopedic percentile between 76 and 100%, 68/174 [39.1%] between 51 and 75%, 26/174 [14.9%] between 26 and 50% and 13/174 [7.5%] between 0 and 25%.

Mixed orthopedic and soft tissue surgery was the major disciplinary focus mentioned by participants (72/174 [41.4%]), followed by orthopedic surgery (45/174 [25.9%]), sports medicine and rehabilitation (39/174 [22.4%]), general practice (13/174 [7.5%]) and other (5/174 [2.9%]). Within the “other” category, the following disciplinary focuses were mentioned: integrative general practice (*n* = 1); mixed-general practice and rehabilitation (*n* = 1); mixed-sports medicine/rehabilitation and orthopedic surgery (*n* = 1); mixed-acupuncture and rehabilitation (*n* = 1); and mixed-rehabilitation, general practice and hospice (*n* = 1).

Most practitioners had their workplace setting in private practice (130/174 [74.7%]), followed by academia (25/174 [14.4%]) and mobile practice (19/174 [10.9%]).

The use of NSIATs was not influenced by the canine orthopedic percentile (*p* = 0.63), major disciplinary focus (*p* = 0.42), workplace setting (*p* = 0.32), or year of graduation (*p* = 0.51).

**Figure 1:**
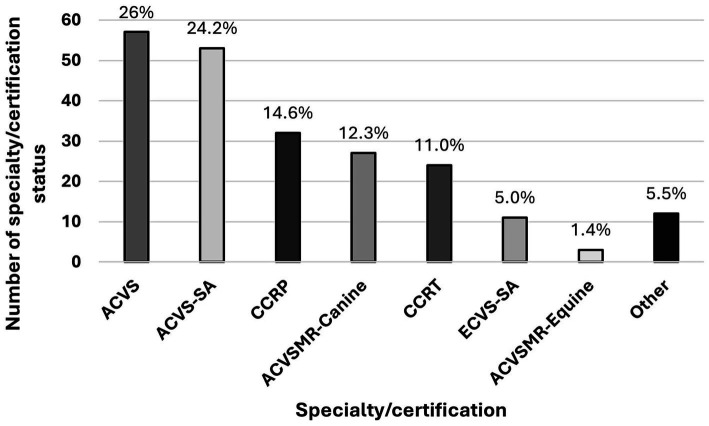
Specialty and/or certification status represented among survey participants. Most of the practitioners (140/174 [80.5%]) had one specialization or certification status, and 34/174 [19.5%] had more than one. In total, 219 specializations and certifications were mentioned by the participants. ACVS was the most common (57/219 [26%]), followed by ACVS-SA (53/219 [24.2%]), CCRP (32/219 [14.6%]), ACVSMR – Canine (27/219 [12.3%]), CCRT (24/219 [11.0%]), ECVS – Small Animal (SA) (11/219 [5.0%]), and ACVSMR – Equine with clinical exposure to dogs (3/219 [1.4%]). Other specializations and certifications (ACVS- Large Animal with clinical exposure to dogs, ECVSMR – Small Animal, ECVSMR – Large Animal with clinical exposure to dogs, CCAT, Certified Veterinary Massage and Rehabilitation Therapy, American Veterinary Chiropractic Association, and Certified Small Animal Rehabilitation Practitioner of the University of Ghent) represented less than 1% each. European College of Veterinary Surgeons (ECVS) – Large Animal was not mentioned by the participants.

One of the inclusion criteria for participating in this survey was having a specialty board status (ACVS, ECVS, ACVSMR, ECVSMR) and/or certification in animal rehabilitation. Participants were able to select all areas of specialization and/or certification status that applied. Most of the practitioners (140/174 [80.5%]) had one specialization or certification status, and 34/174 [19.5%] had more than one. The use of NSIATs was not influenced by whether the practitioner had a single or multiple specialization or certification status (*p* = 0.07). In total, the participants mentioned 219 specializations and certifications (156 specializations and 63 certification statuses). ACVS, ACVS-Small Animal (SA), and CCRP were the most represented specialty boards and certifications among practitioners (Figure 1). There was no difference in NSIATs use and year of specialty status (*p* = 0.11) or certification status (*p* = 0.20).

### Geographic location

Most participants practiced in the United States of America (USA) (151/174 [86.8%]). Of the USA surveyed participants, 40/174 [23.0%] practiced in the southeast, 34/174 [19.5%] in the west, 33/174 [19.0%] in the northeast, 32/174 [18.4%] in the midwest, and 12/174 [6.9%] in the southwest. There were no differences among participants’ geographic locations inside the USA (*p* > 0.005). The rest of the participants practiced internationally (23/174 [13.2%]) including practitioners from Canada (*n* = 11), United Kingdom (*n* = 2), New Zealand (*n* = 2), Australia (*n* = 2), Hong Kong (*n* = 2), Finland (*n* = 1), Belgium (*n* = 1), Portugal (*n* = 1) and Ireland (*n* = 1). Participants who practiced in the USA were more likely to use NSIATs than non-USA participants (*p* = 0.006).

### Overall usage of intra-articular injections

**Figure 2:**
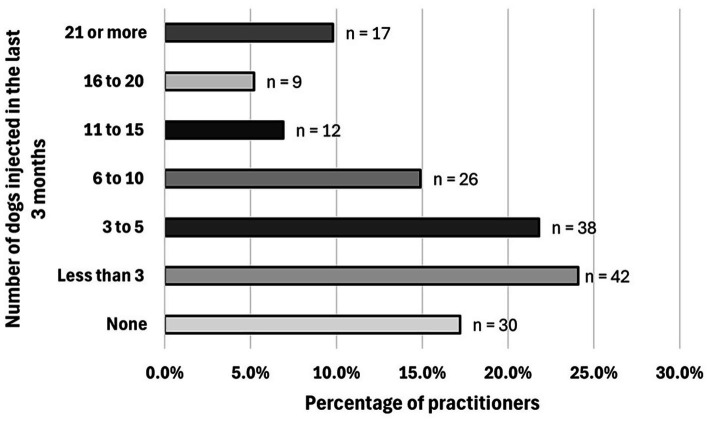
Response count of the number of dogs injected intra-articularly in the last 3 months by veterinary practitioners. Total number of participants who responded: 174 practitioners.

**Figure 3:**
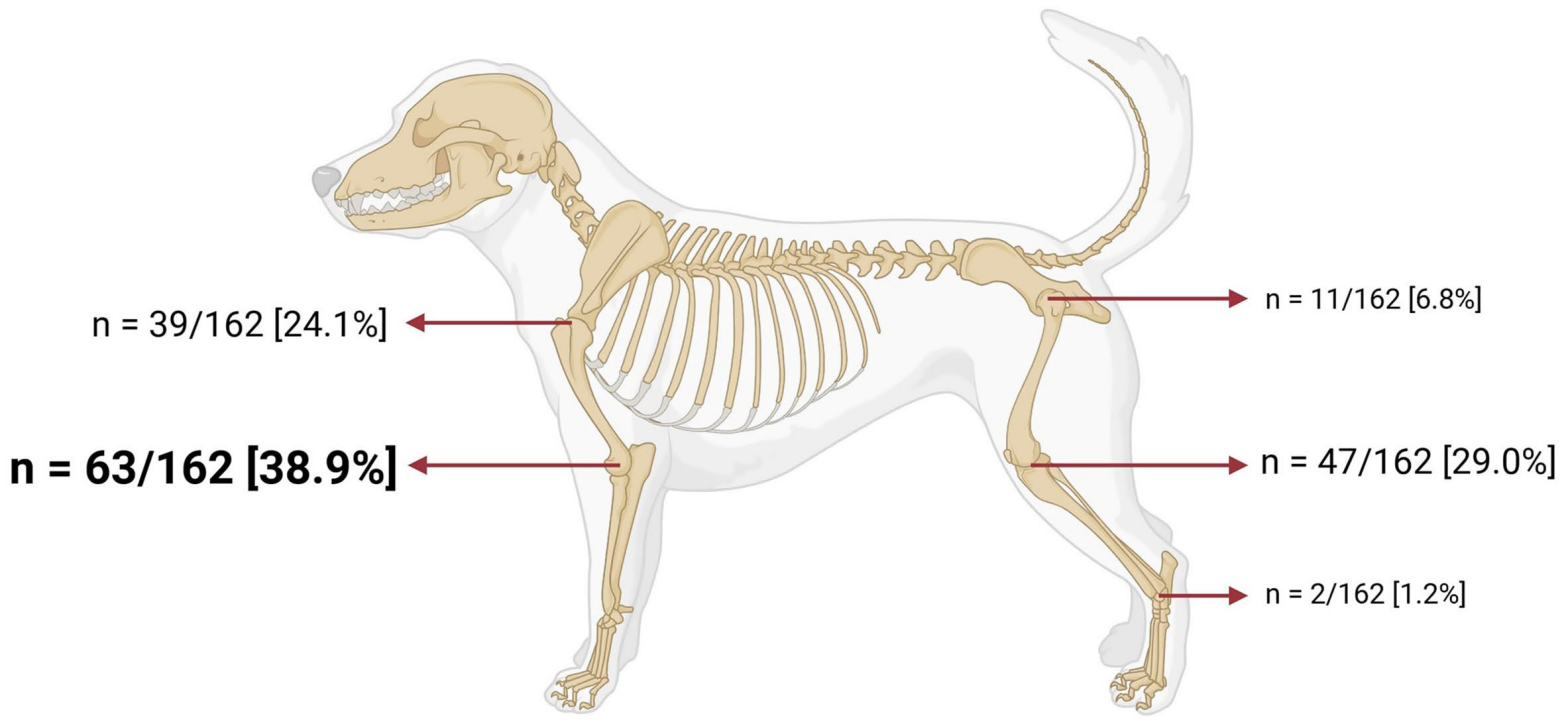
Joint most injected intra-articularly by veterinary practitioners. Total number of participants who responded: 162 practitioners. This figure was created using Biorender.com.

Practitioners were asked to mention, on average, how many dogs they performed IA injections (steroidal and non-steroidal IA therapies) in the last 3 months. Thirty of 174 [17.2%] did not perform joint injections, 42/174 [24.1%] injected less than 3 dogs, 38/174 [21.8%] injected between 3 to 5 dogs, 26/174 [14.9%] injected between 6 to 10 dogs, 12/174 [6.9%] injected between 11 to 15 dogs, 9/174 [5.2%] injected between 16 and 20 dogs, and 17/174 [9.8%] injected 21 dogs or more (Figure 2). Participants who did not perform any IA injections in the last 3 months were less likely to use NSIATs (*p* < 0.0001) compared to other groups. Participants were asked to report the most common joint in which they use IA injections. This question was eliminated from 12 surveys due to the following reasons: inconsistencies in the answer (*n* = 2) and practitioners not performing IA injections (*n* = 10). One hundred sixty-two responses were included. Sixty-three of 162 [38.9%] participants reported that the most common joint injected was the elbow, 47/162 [29.0%] followed by stifle, 39/162 [24.1%] shoulder, 11/162 [6.8%] hip, and 2/162 [1.2%] tarsus. The carpus was not reported as a common location for IA injections (Figure 3).

Of the 164 participants who performed IA injections, 144/164 [87.8%] used NSIATs, while 20/164 [12.2%] did not use these products.

Practitioners who do not use NSIATs were asked to mention at least two main reasons for not using these types of therapeutics. Table 2 shows the participants’ first-choice reasons and second-choice reasons for not using NSIATs in their practices. One practitioner selected the “other” option to report that being a locum surgeon was the first-choice reason for not using NSIATs. Three practitioners selected the “other” option to mention the use of sedatives (*n* = 1), enough orthopedic specialists in the area offering the service (*n* = 1), and lack of follow-through (*n* = 1) as other second-choice reasons for not using NSIATs.

**Table 2:** Participants’ reasons for not using NSIATs.

Reasons for not using NSIATs in their practice	First-choice reason	Second-choice reason
Unclear benefits in current literature	11 [55.0%]	2 [10.0%]
Lack of training	2 [10.0%]	2 [10.0%]
Lack of personal clinical experience	3 [15.0%]	4 [20.0%]
Cost of product or instrumentation	3 [15.0%]	5 [25.0%]
Lack of product availability	0	4 [20.0%]
Lack of client interest	0	0
Other #1 - Please specify	1 [5.0%]	2 [10.0%]
Other #2 - Please specify	0	1 [5.0%]

Number of participants who selected first-choice reason and second-choice reason for not using NSIATs in their practice. Total number of participants who responded: 20 practitioners.

### Overall usage of NSIATs

**Figure 4:**
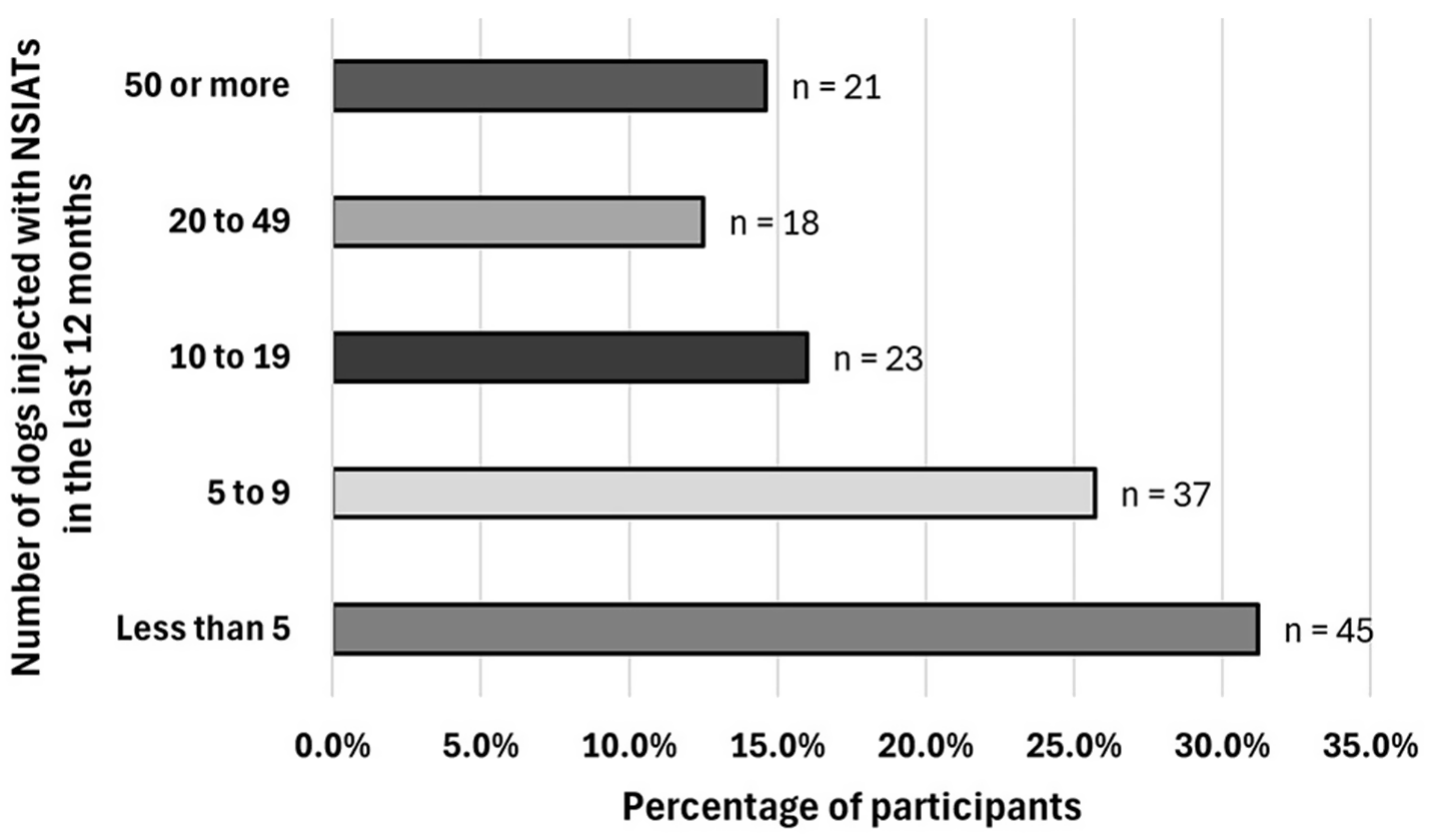
Number of dogs injected with NSIATs in the last 12 months. Total number of participants who responded: 144 practitioners.

Participants were asked to report, on average, the number of dogs they have injected with NSIATs in the last 12 months. Forty-five of 144 [31.2%] practitioners estimated using NSIATs in less than 5 dogs, 37/144 [25.7%] used NSIATs in 5 to 9 dogs, 23/144 [16.0%] injected NSIATs in 10 to 19 dogs, 18/144 [12.5%] used NSIATs in 20 to 49 dogs, and 21/144 [14.6%] injected NSIATs in 50 dogs or more in the last 12 months (Figure 4).

Practitioners were asked to mention the top three reasons for their decision as to which NSIATs they preferentially use. The participants’ most common first-choice reason was scientific data and articles published regarding the product’s safety and efficacy (68/144 [47.2%]). Table 3 summarizes the reasons provided by participants for selecting NSIATs. One practitioner selected the “other” option to mention the absence of other treatment options as a first-choice reason. One practitioner selected the “other” option to mention continued education seminars as a second-choice reason. Four practitioners selected the “other” option to mention: what is used in surgical internship and residency training (*n* = 1), regenerative medicine goals (*n* = 1), age of the dog (*n* = 1), and limited other options (*n* = 1) as third-choice reasons.

**Table 3:** Participants’ reasons for selecting NSIATs.

Reasons for NSIATs selection	First-choice reason	Second-choice reason	Third-choice reason
Scientific data and articles published regarding the product’s safety and efficacy	68 [47.2%]	37 [25.7%]	11 [7.6%]
Personal experience with the product	40 [27.8%]	43 [29.9%]	24 [16.7%]
Availability of the product	4 [2.8%]	13 [9.0%]	30 [20.8%]
The specific joint being treated	0	8 [5.6%]	18 [12.5%]
Cost of product	0	15 [10.4%]	24 [16.7%]
Specific condition being treated	30 [20.8%]	26 [18.1%]	26 [18.1%]
Client request	1 [0.7%]	1 [0.7%]	7 [4.9%]
Other - Please specify	1 [0.7%]	1 [0.7%]	4 [2.8%]

Number of participants who selected first-choice reason, second-choice reason, and third-choice reason for their decision as to which NSIATs they preferentially use. Total number of participants who responded: 144 practitioners.

**Figure 5:**
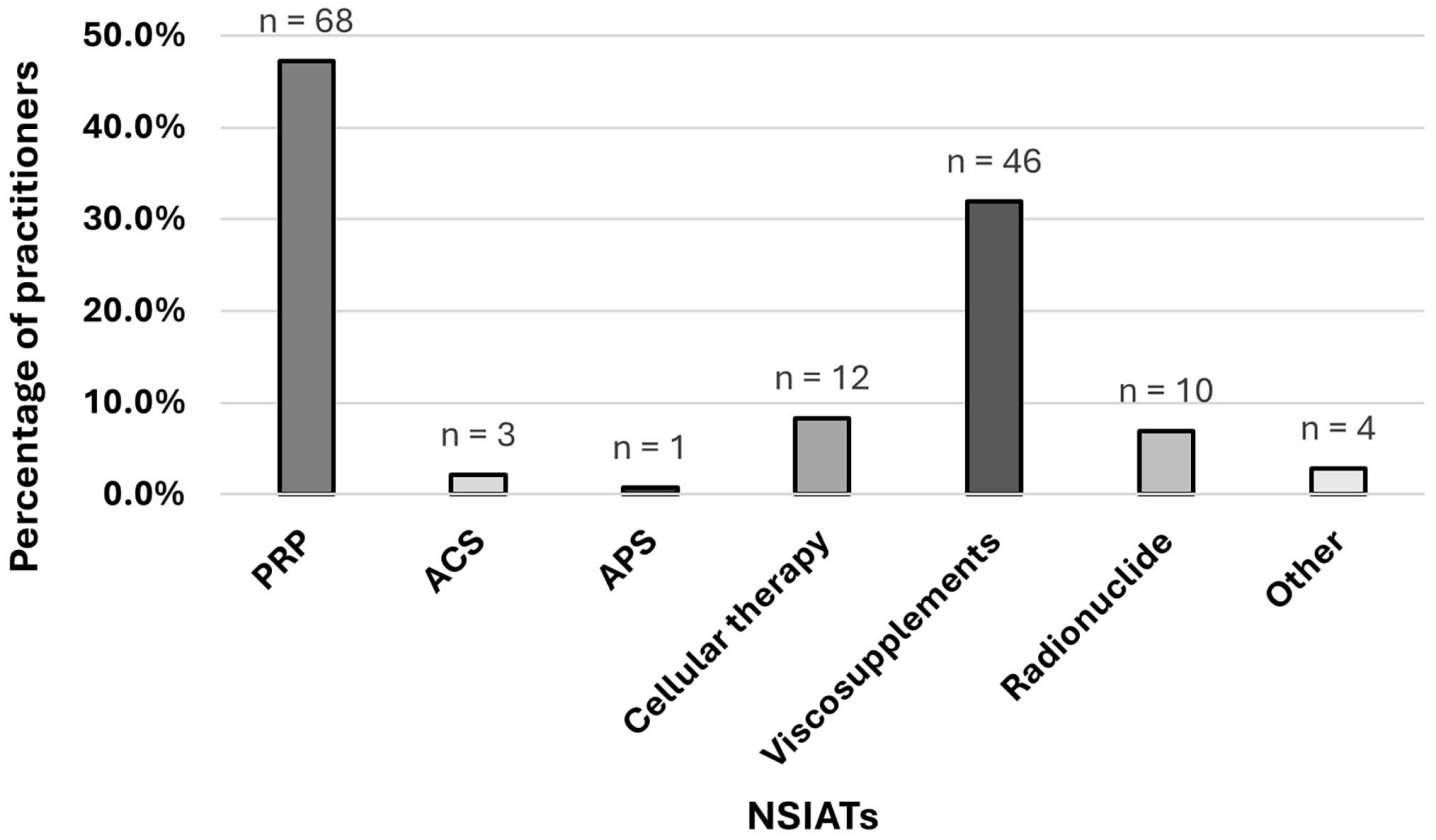
Veterinary practitioner preferred NSIAT (regardless of client preference and product availability). Total number of participants who responded: 144 practitioners.

Participants were asked to mention their preferred NSIATs (regardless of client preference or product availability) when treating joint-related conditions in dogs. Platelet-rich plasma was the top choice (68/144 [47.2%]), followed by viscosupplements (46/144 [31.9%]), cellular therapy (12/144 [8.3%]), radionuclides (10/144 [6.9%]), ACS (3/144 [2.1%]), and APS (1/144 [0.7%]). Four of 144 [2.8%] practitioners specified other options as their first choice, mentioning dextrose (*n* = 2), ozone hydrogen (*n* = 1), and that the preference depended on the specific condition being treated (*n* = 1) (Figure 5). Practitioners who preferred cellular therapy over other therapies tended to inject less than 20 dogs in the last 12 months (*p* = 0.03). Statistical analysis was similarly performed for all other combinations, and no statistically significant differences were observed.

### Platelet-rich plasma (PRP) or autologous conditioned plasma (ACP)

Survey results for PRP or ACP therapy (where ACP represents a specific method of processing PRP that results in lower platelet enrichment compared to PRP) are summarized in Table 4. Of the 144 participants who used NSIATs, 116 [80.6%] used PRP or ACP, while 28 [19.4%] did not.

**Table 4:** Summary of each NSIATs included in the survey from a total of 144 participants to the survey.

Categories of evaluation	PRP	ACS	APS	Cellular therapy	Viscosupplements	Radionuclide
Number of practitioners using this product	116/144; 80.6%	4/144; 2.8%	3/144; 2.1%	43/144; 29.9%	112/144; 77.8%	24/144; 16.7%
Most common reason for practitioner use of the product	Chronic articular pathology needing ‘maintenance’ or routine injections (52/116 [44.8%])	Chronic articular pathology needing ‘maintenance’ or routine injections (3/4 [75.0%])	Acute articular pathology (3/3 [100%])	Chronic articular pathology needing ‘maintenance’ or routine injections (24/43 [55.8%])	Chronic articular pathology needing ‘maintenance’ or routine injections (72/112 [64.3%])	Chronic articular pathology needing ‘maintenance’ or routine injections (22/24 [91.7%])
Most mentioned product used in combination for IA injection	None (66/116 [56.9%])	None (3/4 [75.0%])	None (2/3 [66.7%])	PRP (25/43 [58.1%])	Corticosteroids (45/112 [40.1%])	None (24/24 [100%])
Most common treatment protocol	Repeated injection based on short-term clinical response (40/116 [34.5%])	One time injection (2/4 [50%]) and repeated injection based on short-term clinical response (2/4 [50%])	One time injection [33.3%]; repeated injection based on short-term clinical response [33.3%] and repeated injection based on long-term clinical response [33.3%]	One time injection (18/43 [41.9%])	One time injection (36/112 [32.1%])	Once per 12-month interval (23/24 [95.8%])

Of the 116 practitioners who used PRP or ACP, 88 participants [75.9%] had a single specialization or certification, while 28 participants [24.1%] had multiple specializations and/or certifications.

Platelet-rich plasma or ACP therapy was mainly performed by practitioners whose major disciplinary focus is mixed orthopedic and soft tissue surgery (43/116 [37.1%]) followed by sports medicine and rehabilitation (31/116 [26.7%]), orthopedic surgery (28/116 [24.1%]), general practice (10/116 [8.6%]), and other (4/116 [3.4%]).

Ninety-two of 116 [79.3%] practitioners who used PRP or ACP have their workplace setting in private practice, 16/116 [13.8%] were in academia, and 8/116 [6.9%] were in mobile practice. Practitioners who work in private practice were more likely to use PRP or ACP than practitioners who work in mobile practice (*p* = 0.02).

Most participants that used PRP or ACP in the USA have their practice located in southeast (28/116 [24.1%]), midwest (27/116 [23.3%]), and west (26/116 [22.4%]), followed by northeast (21/116 [18.1%]) and southwest (5/116 [4.3%]). Nine of 116 [7.8%] practitioners who used PRP or ACP in their practice were non-USA residents.

The use of PRP or ACP was not influenced by canine orthopedic percentile (*p* = 0.31), major disciplinary focus (*p* = 0.09), participants’ geographic location (USA residents vs. non-USA residents) (*p* = 0.14), or year of graduation (*p* = 0.52).

Practitioners were asked to mention how the PRP or ACP is processed in their practice prior to administration. One hundred six of 116 [91.4%] participants reported using centrifugation with a commercialized kit and centrifuge, 6/116 [5.2%] used manual centrifugation, and 2/116 [1.7%] mentioned relying on an outside laboratory or referral center for PRP processing. Additionally, 2/116 [1.7%] practitioners indicated that they do not process PRP. Instead, they used a freeze-dried product reconstituted with sterile water (i.e., PrecisePRP™). None of the participants mentioned filtration as a processing method prior to PRP administration.

**Figure 6:**
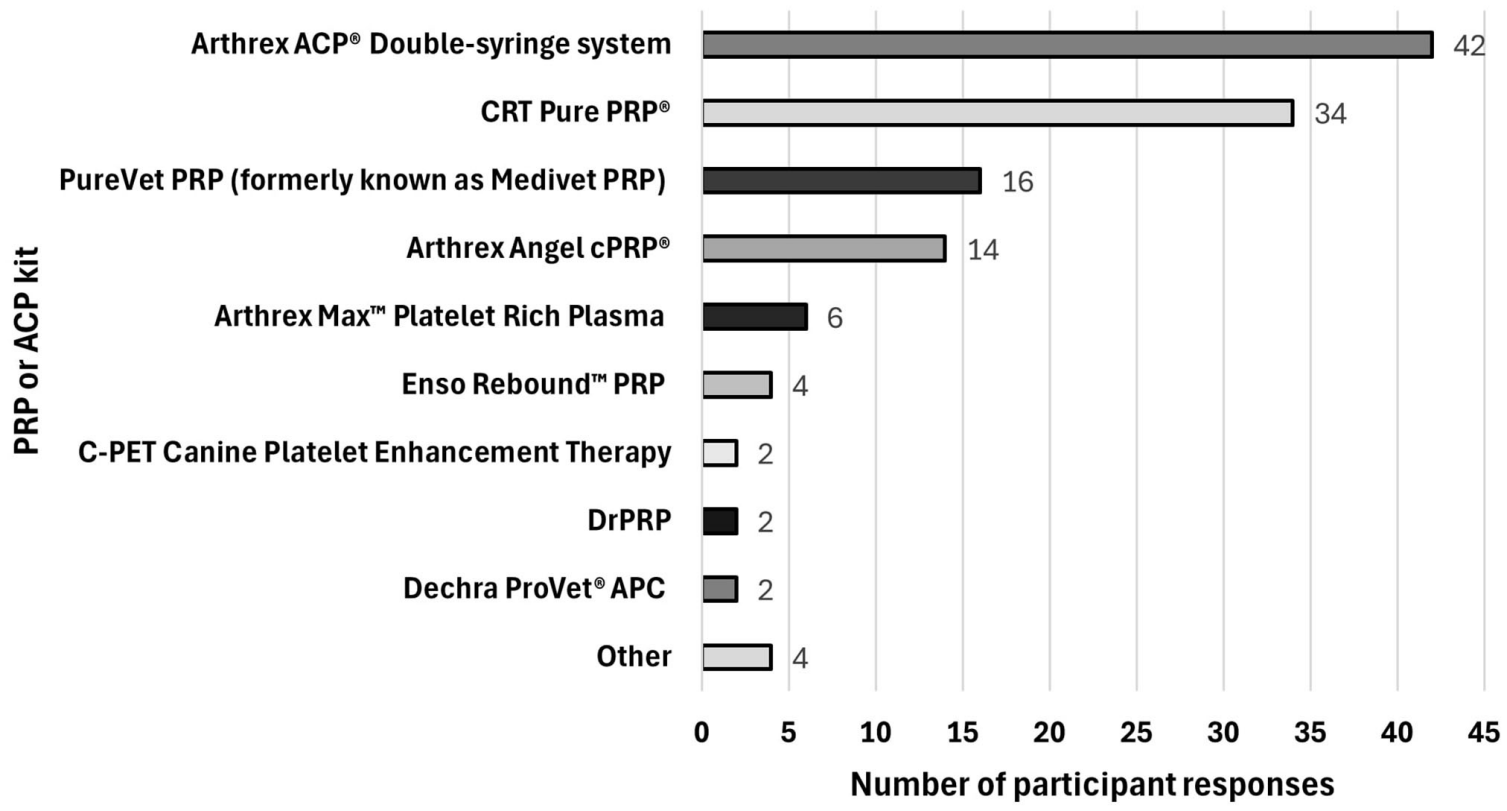
PRP or ACP commercial kits used by veterinary practitioners. Arthrex ACP®️ double-syringe system (42/126 [33.3%]) and CRT Pure PRP®️ (34/126 [27.0%]) were the commercial kits most commonly used by practitioners, followed by PureVet PRP (formerly known as Medivet PRP) (16/126 [12.7%]), Arthrex Angel cPRP®️ (14/126 [11.1%]), Arthrex Max™ Platelet Rich Plasma (6/126 [4.8%]), and C-PET Canine Platelet Enhancement Therapy (2/126 [1.6%]). Twelve/126 [9.5%] other kits, not included within the proposed choices, were mentioned by the participants: Enso Rebound™ PRP (*n* = 4), DrPRP (*n* = 2), Dechra ProVet®️ APC (*n* = 2), InGeneron (*n* = 1), RegenPRP®️ (*n* = 1), Emcyte PurePRP®️ (*n* = 1), and a kit from Virginia Tech Marion duPont Scott Equine Medical Center (*n* = 1).

Participants were asked to mention what commercial kit(s) they used to process PRP or ACP. Participants were able to select all commercial kit(s) that applied. One hundred twenty-six kits were selected by the practitioners. Arthrex ACP® Double-syringe system (42/126 [33.3%]) and CRT Pure PRP® (34/126 [27.0%]) were the commercial kits most commonly used by practitioners. Figure 6 summarizes the commercial kits reported by the participants. Kits mentioned in the survey but not selected by the participants were: ProTec PRP, Pulse Veterinary Technologies; Restigen®PRP, Zoetis; SmartPReP®ACP + System, Harvest Technologies; Stryker RegenKit®, Regen Lab USA and TropoVet™ PRP, Estar Medical. Seven of 116 [6.0%] practitioners mentioned that they do not use any kit.

Regarding the activation agent, 51/116 [44.0%] participants did not activate their PRP or ACP before administration. Among the remaining respondents, 30/116 [25.9%] participants mentioned physiologic or endogenous activation (allowing the joint environment to activate the platelets), 16/116 [13.8%] used extracorporeal shockwave for activation, 7/116 [6.0%] used calcium chloride, 6/116 [5.2%] used calcium gluconate, and 1/116 [0.9%] mentioned using freeze/thaw cycles. Five of 116 practitioners [4.3%] selected the “other” option to mention different activation methods, which included physiologic stimulation or extracorporeal shockwave depending on the condition treated (*n* = 1), physiologic stimulation and extracorporeal shockwave (*n* = 1), the activation agent that the system had (*n* = 1), citrate dextrose (*n* = 1), and unknown information (*n* = 1).

Sixty-three of 116 [54.3%] practitioners reported using concurrent oral or systemic injectable non-steroidal anti-inflammatory drug (NSAID), while 53/116 [45.7%] did not concurrently administer NSAIDs.

Participants were asked if they ensured that the dog was not on any specific medications prior to pulling and processing PRP or ACP. If the answer was yes, they were then asked to specify which drugs. Thirty-four of 116 [29.3%] practitioners ensured that the dog was not on any medications. Medications mentioned included: NSAIDs (*n* = 20), corticosteroids (*n* = 5), NSAIDs and corticosteroids (*n* = 4), anticoagulants (*n* = 4), and clopidogrel and aspirin (*n* = 1). Eighty-two of 116 [70.7%] practitioners did not ensure if the patient was on specific drugs prior to pulling and processing PRP or ACP.

Practitioners were asked to mention the top two most common reasons they use PRP or ACP in their patients. As the first most common reason, 52/116 [44.8%] participants mentioned chronic articular pathology needing ‘maintenance’ or routine injections, followed by ligament or tendon lesions (38/116 [32.8%]), acute articular pathology (15/116 [12.9%]), postoperative therapy (8/116 [6.9%]), tendon sheath or bursa applications (2/116 [1.7%]) and preventative or prophylactic measure (1/116 [0.9%]). As the second most common reason, 39/116 [33.6%] participants mentioned ligament or tendon lesions, followed by tendon sheath or bursa applications (25/116 [21.6%]), postoperative therapy (21/116 [18.1%]), chronic articular pathology needing ‘maintenance’ or routine injections (15/116 [12.9%]), acute articular pathology (14/116 [12.1%]), and preventative or prophylactic measure (2/116 [1.7%]).

Participants were asked if they simultaneously administered other IA products with PRP. Most participants did not combine PRP or ACP with other IA medications or products (66/116 [56.9%]). Thirty-five of 116 [30.2%] practitioners used viscosupplements with PRP or ACP, 6/116 [5.2%] practitioners combined PRP or ACP with cellular therapy, 4/116 [3.4%] used antibiotics, with gentamicin mentioned in all cases (dose was mentioned in one case:1–2 mg/kg) and 1/116 practitioner [0.9%] used corticosteroids (triamcinolone, dose not specified). Four of 116 [3.4%] practitioners selected the “other” option to mention specifically how they combine products: PRP or ACP with hyaluronic acid (HA) and/or triamcinolone (3–5 mg/joint) (*n* = 1); PRP or ACP with stem cells and HA (*n* = 1); PRP or ACP with HA or Noltrex®Vet or cellular therapy (*n* = 1); PRP or ACP with HA or triamcinolone (4–6 mg/joint) (*n* = 1).

Practitioners were asked to mention their typical treatment protocol with PRP or ACP. Forty of 116 [34.5%] participants repeated injection based on short-term clinical response (i.e., re-injection performed within 3 months of initial therapy), 31/116 [26.7%] participants repeated injection every 1–2 weeks for 3 treatments, 18/116 [15.5%] practitioners repeated injection based on long-term clinical response (i.e., ‘maintenance’ therapy performed every 6 months–1 year), and 12/116 [10.3%] practitioners used PRP or ACP as a one-time injection. Fifteen of 116 participants selected the “other” option to specify their personalized protocols: it depends on the condition being treated (*n* = 3); 1 or 2 injections depending on response (*n* = 2); every 4 weeks for 3 treatments (*n* = 1); every 3 weeks for 3 treatments (*n* = 1); every 2 weeks if joint effusion is still present (*n* = 1); every 3–4 weeks for 3 treatments (*n* = 1); every 2 weeks for 2–3 treatments (*n* = 1); repeat injection 3 weeks later (*n* = 1); repeat injection 4 weeks later (*n* = 1); every 6–24 months as needed (*n* = 1); repeat injection at 2–3 weeks, at 6 months and every 6–12 months (*n* = 1); usually a one-time thing but few patients have had repeat injections over the years (*n* = 1).

**Figure 7:**
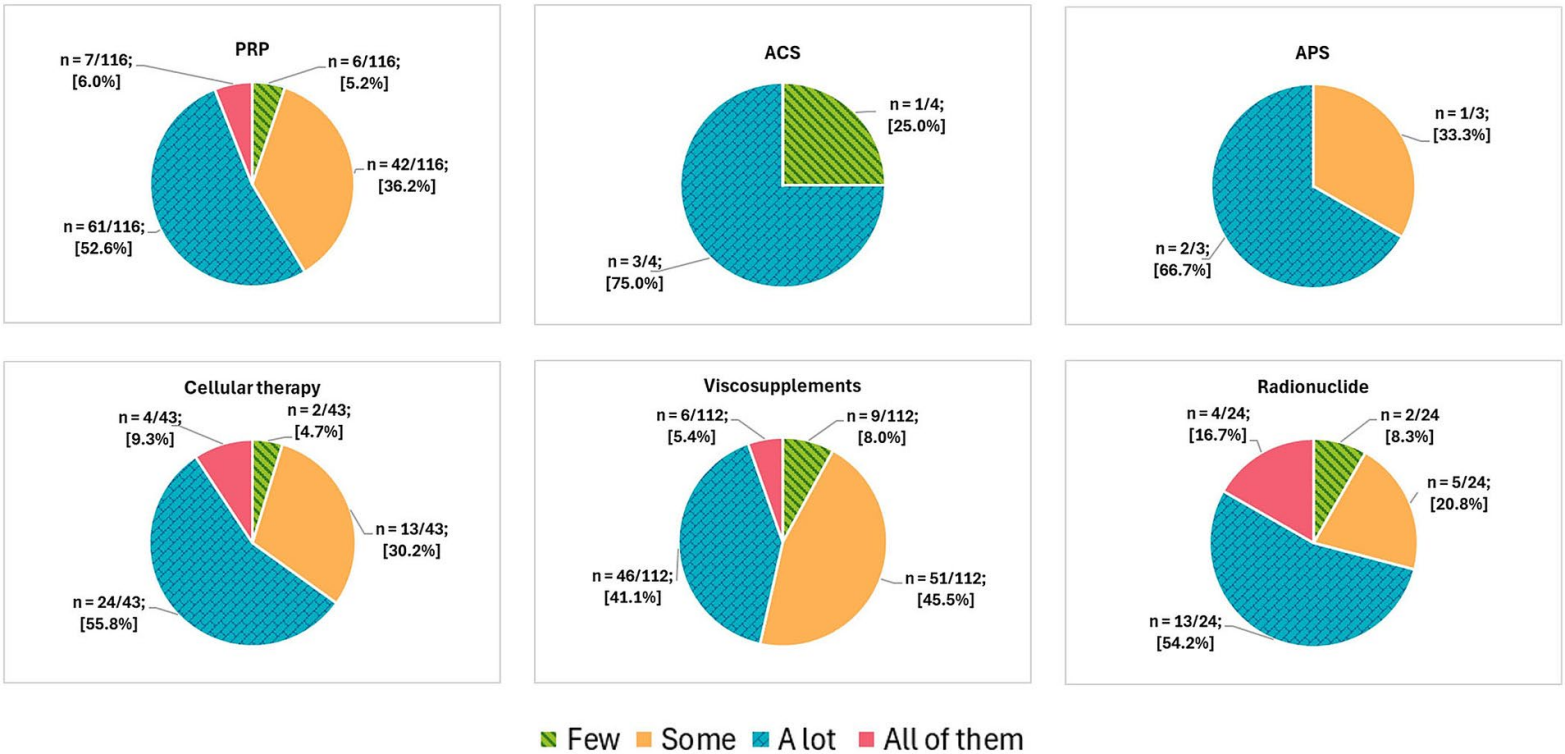
Practitioners’ perceived proportion of case responders to NSIAT administration. Participants were asked to subjectively mention one of the following options: few, some, a lot, or all of them. This figure was created using Biorender.com.

Sixty-one of 116 [52.6%] participants indicated that “a lot” of their cases seemed to respond after PRP or ACP administration, 42/116 [36.2%] practitioners mentioned that “some” of their patients seemed to respond, 7/116 [6.0%] practitioners reported that “all” of their cases seemed to respond and 6/116 [5.2%] participants reported that “few” patients seemed to respond to treatment (Figure 7). There were no differences in perceived proportion of case responders between the use of PRP alone and its association with viscosupplements (*p* > 0.99). Statistical analysis was similarly performed for all other combinations, and no statistically significant differences were observed.

**Figure 8:**
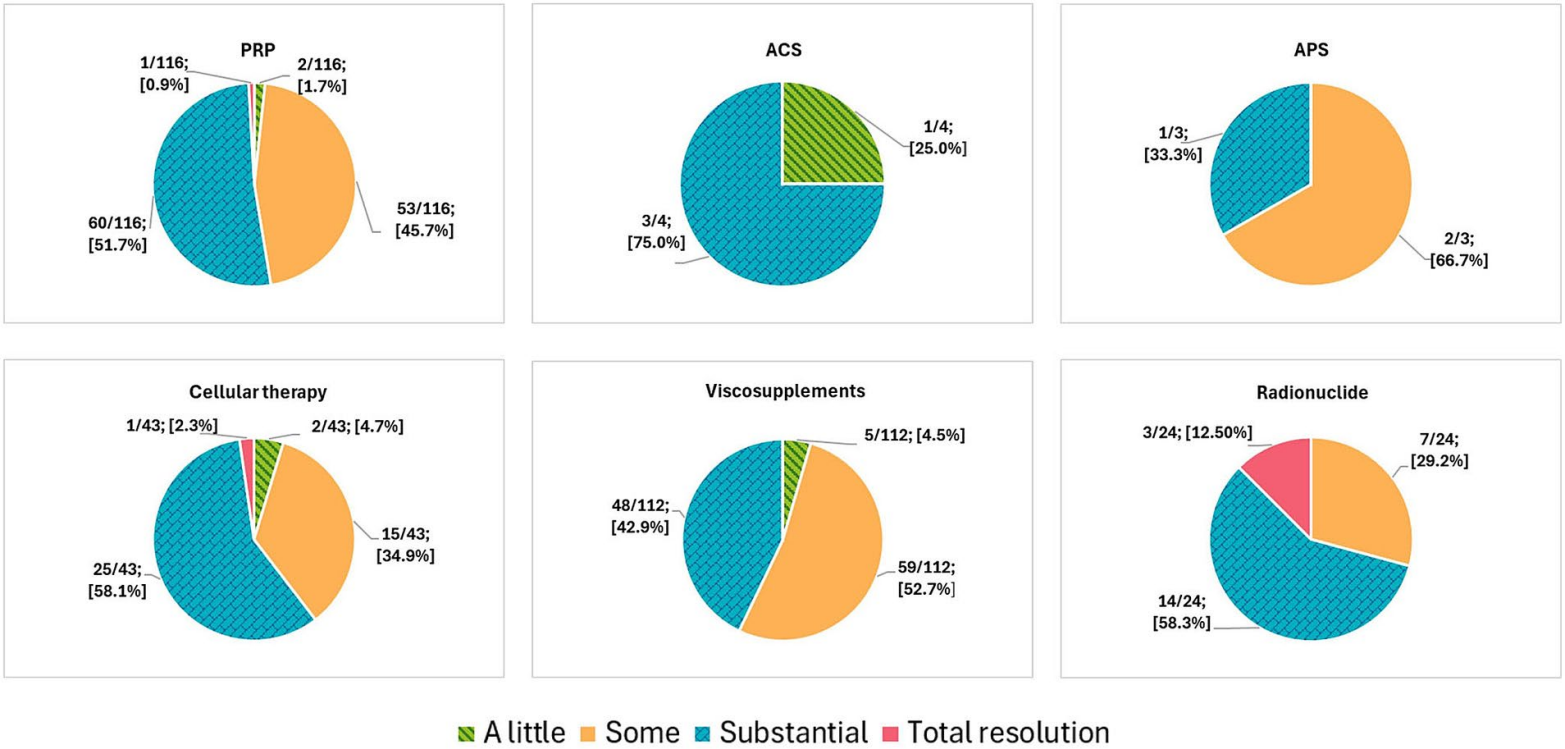
Practitioners’ perceptions of clinical improvement after NSIAT use. Number of practitioners who mentioned how much clinical improvement they tend to see in their positive responders’ cases after NSIAT administration. Participants were asked to subjectively mention one of the following options: a little, some, substantial, or total resolution. This figure was created using Biorender.com.

Regarding those positive responders, 60/116 [51.7%] participants estimated that their patients had “substantial” clinical improvement, 53/116 [45.7%] reported “some” clinical improvement, 2/116 [1.7%] reported “a little” clinical improvement, and 1/116 [0.9%] practitioner reported “total resolution” (Figure 8). There were no differences in perceived clinical improvement between the use of PRP alone and its association with viscosupplements (*p* = 0.67). Statistical analysis was similarly performed for all other combinations, and no statistically significant differences were observed.

There was no difference in perceived outcomes between PRP processing methods. Regarding PRP activation, participants who did not use an activation agent or allowed endogenous activation were more likely to perceive a greater clinical improvement (substantial/total resolution) than those who used calcium gluconate (*p* = 0.02). Similarly, participants who used extracorporeal shockwave were more likely to perceive a greater clinical improvement (substantial/total resolution) than those who used calcium gluconate (*p* = 0.002). Statistical analysis was similarly performed for all other PRP activation methods, and no statistically significant differences were observed.

Practitioners were asked about the incidence of acute joint flares they encountered in their patients after IA injection with PRP or ACP. A joint flare was defined as a period of increased disease activity or worsening of clinical signs in a particular joint (e.g., increased joint pain, inflammation, and lameness). Over 50% of the participants reported observing joint flare in 5% or fewer of their patients. Survey results for the incidence of acute joint flares are summarized in Table 5.

**Table 5:** Incidence of acute joint flares reported across all NSIATs evaluated in the survey, expressed as the number of cases relative to total users (incidence/total users).

Incidence of acute joint flare	PRP	ACS	APS	Cellular therapeutics	Viscosupplements	Radionuclide
None	28/116 [24.1%]	1/4 [25.0%]	1/3 [33.3%]	16/43 [37.2%]	59/116 [52.7%]	5/24 [20.8%]
1 in 50 dogs (2% of dogs)	29/116 [25.0%]	1/4 [25.0%]	1/3 [33.3%]	12/43 [27.9%]	29/112 [25.9%]	5/24 [20.8%]
1 in 20 dogs (5% of dogs)	13/116 [11.2%]	1/4 [25.0%]		1/43 [2.3%]	10/112 [8.9%]	3/24 [12.5%]
1 in 10 dogs (10% of dogs)	24/116 [20.7%]			10/43 [23.3%]	4/112 [3.6%]	2/24 [8.3%]
1 in 5 dogs (20% of dogs)	15/116 [12.9%]		1/3 [33.3%]	1/43 [2.3%]	5/112 [4.5%]	1/24 [4.2%]
1 in 2 dogs (50% of dogs)	6/116 [5.2%]	1/4 [25.0%]		1/43 [2.3%]	4/112 [3.6%]	7/24 [29.2%]
All cases	1/116 [0.9%]			2/43 [4.7%]	1/112 [0.9%]	1/24 [4.2%]

### Autologous conditioned serum (ACS)

Survey results for ACS therapy are summarized in Table 4. Of the 144 participants that used NSIATs, 4 [2.8%] used ACS while 140 [97.2%] did not.

Of the 4 practitioners who used ACS, 1 participant [25.0%] had a single specialization or certification, while 3 participants [75.0%] had multiple specializations and/or certifications.

Autologous conditioned serum therapy was performed by practitioners whose major disciplinary focus was sports medicine and rehabilitation (2/4 [50.0%]) and orthopedic surgery (2/4 [50.0%]).

Two of 4 [50.0%] practitioners who used ACS had their workplace setting in private practice, and 2/4 [50.0%] in academia.

All participants who used ACS in the USA had their practice located in the southeast (*n* = 3). One of 4 [25.0%] practitioners who used ACS was a non-USA resident.

Due to the small sample size, the previous data was not subjected to statistical analysis.

Participants were asked to specify what ACS commercial kit they used. Two of 4 [50.0%] participants mentioned Dechra Orthokine®Vet IRAP 10, 1/4 [25.0%] practitioners mentioned Arthrex Orthobiologics IRAP II™ Systems, and 1/4 [25.0%] practitioners used Orthogen® Device Vet.

Practitioners were asked to mention the top two most common reasons they use ACS in their patients. As the first most common reason, 3/4 [75.0%] participants mentioned chronic articular pathology needing ‘maintenance’ or routine injections, followed by postoperative therapy (1/4 [25.0%]). As the second most common reason, 2/4 [50.0%] participants mentioned acute articular pathology, followed by preventative or prophylactic measures (1/4 [25%]). One of 4 [25.0%] practitioners selected the “other” option to report that there was no second reason for using ACS.

Participants were asked if they simultaneously administered other IA products with ACS. Most participants (3/4 [75.0%]) did not combine ACS with other IA medications or products. One of 4 [25.0%] practitioners used viscosupplements with ACS.

Practitioners were asked to mention their typical treatment protocol for ACS. Two of 4 [50.0%] participants performed a one-time injection, while the other half (2/4 [50.0%]) repeated injection based on short-term clinical response (i.e., re-injection performed within 3 months of initial therapy).

Three of 4 [75.0%] participants indicated that “a lot” of patients seemed to respond after ACS therapy, while 1/4 [25.0%] practitioners mentioned that “few” patients seemed to respond to treatment (Figure 7).

Regarding those positive responders, 3/4 [75.0%] practitioners estimated that their patients had “substantial” clinical improvement, while 1/4 [25.0%] practitioners reported “a little” clinical improvement (Figure 8).

A varying incidence of acute joint flare was reported by the participants. Survey results for the incidence of acute joint flares following IA injection with ACS are summarized in Table 5.

### Autologous protein solution (APS)

Survey results for APS therapy are summarized in Table 4. Of the 144 participants that used NSIATs, 3 [2.1%] used APS while 141 [97.9%] did not.

All practitioners who used APS had a single specialization or certification.

Autologous protein solution therapy was performed by practitioners whose major disciplinary focus is orthopedic surgery (2/3 [66.7%]), followed by mixed orthopedic and soft tissue surgery (1/3 [33.3%]).

Two of 3 [66.7%] practitioners who used APS had their workplace setting in private practice, and 1/3 [33.3%] in academia.

Two of 3 [66.7%] participants who used APS in the USA had their practice located in the southeast, and 1/3 [33.3%] in the midwest. None of the practitioners who used APS were non-USA residents.

Due to the small sample size, the previous data was not subjected to statistical analysis.

Practitioners were asked to mention the top two most common reasons they use APS in their patients. As the first most common reason, all [100.0%] participants mentioned acute articular pathology. As the second most common reason, 2/3 [66.7%] participants mentioned ligament or tendon lesions, followed by chronic articular pathology needing ‘maintenance’ or routine injections (1/3 [33.3%]).

Participants were asked if they simultaneously administered other IA products with APS. Two of 3 [66.7%] of participants did not combine APS with other IA medications or products. One of 3 [33.3%] practitioners reported the use of viscosupplements with APS.

Two of 3 [66.7%] of practitioners reported using concurrent oral or systemic injectable NSAID when administering IA APS, while 1/3 [33.3%] participants did not concurrently administer NSAIDs.

Participants were asked if they ensured that the dog was not on any specific medications prior to pulling and processing APS. If the answer was yes, they were then asked to specify which drugs. One of 3 [33.3%] of practitioners ensured that the dog was not on any medications, specifically NSAIDs. Two of 3 [66.7%] practitioners did not ensure if the canine patient was on specific drugs prior to pulling and processing the APS.

Practitioners were asked to mention their typical treatment protocol for APS. The 3 participants provided different protocols. One [33.3%] performed a one-time injection, while the other 2 [33.3% each] repeated injections based on short-term clinical response (i.e., re-injection performed within 3 months of initial therapy) and long-term clinical response (i.e., ‘maintenance’ therapy performed every 6 months–1 year), respectively.

Two of 3 [66.7%] participants indicated that “a lot” of their cases seemed to respond after APS therapy, while 1/3 [33.3%] practitioners mentioned that “some” of their patients seemed to respond to treatment (Figure 7).

Regarding those positive responders, 2/3 [66.7%] practitioners estimated “some” clinical improvement, and 1/3 [33.3%] practitioners reported “substantial” clinical improvement (Figure 8).

A varying incidence of acute joint flare was reported by the participants. Survey results for the incidence of acute joint flares following IA injection with APS are summarized in Table 5.

### Cellular therapeutics

Survey results for cellular therapeutics are summarized in Table 4. Of the 144 participants that used NSIATs, 43 [29.9%] used cellular therapeutics while 101 [70.1%] did not.

Of the 43 practitioners who used cellular therapeutics, 29 participants [67.5%] had a single specialization or certification, while 14 participants [32.5%] had multiple specializations and/or certifications.

Cellular therapy was mainly performed by practitioners whose major disciplinary focus is sports medicine and rehabilitation (17/43 [39.5%]) followed by mixed orthopedic and soft tissue surgery (11/43 [25.6%]), orthopedic surgery (9/43 [20.9%]), general practice (5/43 [11.6%]), and other (1/43 [2.3%]).

Thirty-six of 43 [83.7%] practitioners who used cellular therapeutics had their workplace setting in private practice, 6/43 [14.0%] were in academia, and 1/43 [2.3%] were in mobile practice.

Most participants that used cellular therapeutics in the USA had their practice located in southeast (11/43 [25.6%]), followed by midwest (8/43 [18.6%]), west (8/43 [18.6%]), northeast (7/43 [16.3%]) and southwest (3/43 [7.0%]). Six of 43 [14.0%] practitioners who used cellular therapeutics in their practice were non-USA residents.

Due to the small sample size, the previous data was not subjected to statistical analysis.

Practitioners were asked to mention the top two most common reasons they use cellular therapeutics in their patients. As the first most common reason, 24/43 [55.8%] participants mentioned chronic articular pathology needing ‘maintenance’ or routine injections, followed by ligament or tendon lesions (14/43 [32.6%]), postoperative therapy (2/43 [4.7%]) preventative or prophylactic measure (1/43 [2.3%]), and acute articular pathology (1/43 [2.3%]). One of 43 [2.3%] practitioners selected the “other” option to report that IA pathology, not always acute, was the first most common reason for using cellular therapeutics. As the second most common reason, 11/43 [25.6%] participants mentioned ligament or tendon lesions, followed by tendon sheath or bursa applications (9/43 [20.9%]), postoperative therapy (7/43 [16.3%]), acute articular pathology (6/43 [14.0%]), chronic articular pathology needing ‘maintenance’ or routine injections (5/43 [11.6%]), and preventative or prophylactic measure (4/43 [9.3%]). One of 43 [2.3%] practitioners selected the “other” option to report that there was no additional reason for using cellular therapeutics.

Participants were asked to mention the most common tissue source for obtaining cellular therapeutics. If the tissue source was adipose, practitioners were then asked to specify the harvest site. Twenty-six of 43 [60.5%] practitioners identified adipose tissue as the most common source for cellular therapeutics. The reported harvest sites were: falciform fat (*n* = 14), abdomen (*n* = 4), falciform or subcutaneous (*n* = 2), falciform and/or omentum (*n* = 2), falciform ligament (*n* = 1), perihepatic (*n* = 1), and calcified (*n* = 1). One practitioner did not specify the adipose tissue location. Thirteen of 43 [30.2%] participants identified bone marrow as the most common tissue source. Four of 43 [9.3%] participants selected the “other” option to report their most common tissue source: allogeneic amniotic stem cells (*n* = 1), allogeneic amniotic membrane/tissue allograft (AlphaFlo®) (*n* = 2), and unspecified (*n* = 1). Tissue sources such as peripheral blood and synovial tissues, which were among the choices, were not reported by the participants.

Practitioners were asked to specify the donor source for the cellular therapy they most commonly use. The majority (37/43 [86%]) of participants reported using an autologous donor source (obtained from the same dog in which the product is to be used). Six of 43 [14%] practitioners used allogeneic donor sources (obtained from a different dog than the receiving dog). None of the participants reported using a xenogeneic donor source (obtained from a different species).

VetStem (12/43 [27.9%]) and Acti-stem Therapy, Ardent Animal Health (11/43 [25.6%]) were the commercialized systems, companies or suppliers most commonly used by practitioners, followed by Pure BMC®, Companion Animal Health (8/43 [18.6%]), AniCell Biotech (2/43 [4.7%]) and DogStem® (1/43 [2.3%]). Nine of 43 [20.9%] participants selected the “other” option to report the following system, company, or supplier not included within the proposed choices: AlphaFlo® (*n* = 2), Virginia Tech Marion duPont Scott Equine Medical Center (*n* = 2), Regenerative Cell Specialists (*n* = 2), Cornell University (*n* = 2), and eQcell Inc. (*n* = 1).

Participants were asked if they simultaneously administered other IA products with cellular therapeutics. Twenty-one of 43 [48.8%] participants combined cellular therapy with PRP. Thirteen of 43 [30.2%] practitioners did not combine cellular therapeutics with other IA products. Two of 43 [4.7%] participants combined cellular therapy with antibiotics, with gentamicin being the antibiotic of choice (dose was not specified). One of 43 [2.3%] participants combined cellular therapy with viscosupplements. Six of 43 [14.0%] practitioners selected the “other” option to specify how they combine products: cellular therapy with PRP or HA (*n* = 1), cellular therapy with PRP alone or PRP in combination with HA (*n* = 1), cellular therapy always combined with HA or in some occasions in combination with PRP or corticosteroid or Arthramid® or Spryng™ (*n* = 1), cellular therapy with PRP and gentamicin (1–2 mg/kg) (*n* = 1), cellular therapy with a numbing agent (*n* = 1), and cellular therapy combined in some occasions with HA if volume allows (*n* = 1). When considering “other” responses that mention cellular therapy with PRP, the total number of participants who combine cellular therapy with PRP increases to 25/43 [58.1%].

Practitioners were asked to mention their typical treatment protocol for cellular therapy. Eighteen of 43 [41.9%] participants used cellular therapy as a one-time injection, 13/43 [30.2%] participants repeated injection based on long-term clinical response (i.e., ‘maintenance’ therapy performed every 6 months–1 year), 7/43 [16.3%] practitioners repeated injection based on short-term clinical response (i.e., re-injection performed within 3 months of initial therapy) and 2/43 [4.7%] practitioners repeated injection every 1–2 weeks for 3 treatments. Three of 43 [7.0%] practitioners selected the “other” option to specify their personalized protocols: monthly for 2–3 injections, then as needed (*n* = 1); for chronic cases triamcinolone injection followed by cellular therapy, otherwise repeat injection based on long term clinical response (*n* = 1); and repeat injections based on long term clinical response by recurrence of clinical signs, typically at about 2 years (*n* = 1).

Twenty-four of 43 [55.8%] participants indicated that “a lot” of their cases seemed to respond after cellular therapy administration, 13/43 [30.2%] practitioners mentioned that “some” of their patients seemed to respond, 4/43 [9.3%] practitioners reported that “all” of their cases seemed to respond and 2/43 [4.7%] participants reported that “few” patients seemed to respond to treatment (Figure 7). There were no differences in perceived proportion of case responders between the use of cellular therapy alone and its association with PRP (*p =* 0.46). Statistical analysis was similarly performed for all other combinations, and no statistically significant differences were observed.

Regarding those positive responders, 25/43 [58.1%] participants estimated that their patients had “substantial” clinical improvement, 15/43 [34.9%] practitioners reported “some” clinical improvement, 2/43 [4.7%] participants reported “a little” clinical improvement, and 1/43 [2.3%] practitioners reported “total resolution” (Figure 8). There were no differences in perceived clinical improvement between the use of cellular therapy alone and its association with PRP (*p =* 0.16). Statistical analysis was similarly performed for all other combinations, and no statistically significant differences were observed. Participants using a bone marrow source were more likely to perceive a greater clinical improvement (substantial/total resolution) than those using adipose tissue (*p* = 0.01).

Over 50% of the participants observed joint flare in 2% or fewer of their patients. Survey results for the incidence of acute joint flares following IA injection with cellular therapeutics are summarized in Table 5.

### Viscosupplements

Survey results for viscosupplements therapy are summarized in Table 4. Of the 144 participants that used NSIATs, 112 [77.8%] used viscosupplements, while 32 [22.2%] did not.

Of the 112 practitioners who used viscosupplements, 84 participants [75.0%] had a single specialization or certification, while 28 participants [25.0%] had multiple specializations and/or certifications.

Intra-articular injections of viscosupplements are primarily performed by practitioners whose major disciplinary focus was mixed orthopedic and soft tissue surgery (41/112 [36.6%]). This was followed by orthopedic surgery (31/112 [27.7%]), sports medicine and rehabilitation (31/112 [27.7%]), general practice (7/112 [6.3%]), and other (2/112 [1.8%]).

Eighty-five of 112 [75.9%] practitioners that used viscosupplements have their workplace setting in private practice, 19/112 [17.0%] worked in academia, and 8/112 [7.1%] were in mobile practice. Practitioners who work in academia were more likely to use viscosupplements than practitioners who work in mobile practice (*p* = 0.03).

Participants that used viscosupplements in the USA have their practice located in the southeast (28/112 [25.0%]), followed by the midwest (26/112 [23.2%]), the northeast (23/112 [20.5%]), west (16/112 [14.3%]) and the southwest (8/112 [7.1%]). Eleven of 112 [9.8%] practitioners who used viscosupplements in their practice were non-USA residents.

The use of viscosupplements was not influenced by the canine orthopedic percentile (*p* = 0.14), major disciplinary focus (*p* > 0.005), participants’ geographic location (USA residents vs. non-USA residents) (*p* > 0.99), and year of graduation (*p* = 0.69).

**Figure 9:**
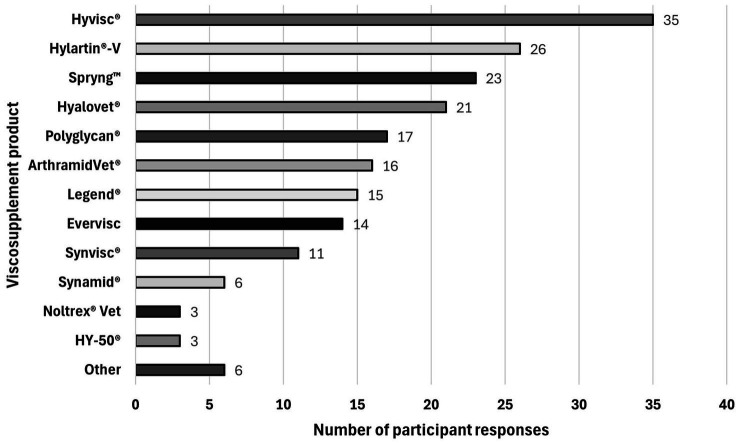
Viscosupplement product used by veterinary practitioners. Hyvisc®️, Boehringer Ingelheim (35/196 [17.9%]) was the viscosupplement most commonly used by practitioners, followed by Hylartin®️-V, Zoetis (26/196 [13.3%]), Spryng™, PetVivo Holdings, Inc. (23/196 [11.7%]), Hyalovet®️, Boehringer Ingelheim (21/196 [10.7%]), Polyglycan®️, SA, Bimeda (17/196 [8.7%]), ArthramidVet®️, Contura Vet (16/196 [8.2%]), Legend®️, Boehringer Ingelheim (15/196 [7.7%]), Synvisc®️ (Hylan G-F 20), Sanofi (11/196 [5.6%]), Synamid®️, Contura Vet (6/196 [3.1%]) and Noltrex®️ Vet, Nucleus ProVets (3/196 [1.5%]). Twenty-three/196 [11.7%] other viscosupplements products, not included within the proposed choices were mentioned by the participants: Evervisc, Movora (*n* = 14), HY-50®️, Dechra (*n* = 3), Hyalur®️, Arthrex (*n* = 2), an-bfn 2.2%, an-vision Inc. (*n* = 1), Hyonate®️, Boehringer Ingelheim (*n* = 1), Adequan®️, American Regent (*n* = 1), and one practitioner indicated that the most commonly used product varies (*n* = 1).

Participants were asked to mention what viscosupplement product(s) they most commonly use. Participants were able to select all the choices that applied. One hundred ninety-six products were selected by the practitioners. Hyvisc®, Boehringer Ingelheim (35/196 [17.9%]), was the most commonly used by practitioners. Figure 9 summarizes the viscosupplement products reported by the participants.

Practitioners were asked to mention the top two most common reasons they use viscosupplements in their patients. As the first most common reason, 72/112 [64.3%] participants mentioned chronic articular pathology needing ‘maintenance’ or routine injections, followed by postoperative therapy (28/112 [25.0%]), acute articular pathology (8/112 [7.1%]), and preventative or prophylactic measure (3/112 [2.7%]). One of 112 [0.95%] practitioners selected the “other” option to indicate that the most common reason for using viscosupplements was to combine it with PRP. As the second most common reason, 37/112 [33.0%] participants mentioned acute articular pathology, followed by postoperative therapy (29/112 [25.9%]), chronic articular pathology needing ‘maintenance’ or routine injections (22/112 [19.6%]), and preventative or prophylactic measure (20/112 [17.9%]). Four of 116 [3.6%] participants selected the “other” option to mention refractory OA (*n* = 1) and no other reasons (*n* = 3).

Participants were asked if they simultaneously administered other IA products with viscosupplements. Forty-two of 112 [37.5%] participants used corticosteroids in conjunction with viscosupplements. Triamcinolone was the most mentioned corticosteroid product (*n* = 28) with doses ranging from 1–6 mg/joint or 0.1–0.5 mg/kg. Methylprednisolone (e.g., Depo-Medrol®) was another corticosteroid mentioned by the participants (*n* = 8), with a dose ranging from 5 to 20 mg/joint. Three participants mentioned the use of triamcinolone or Depo-Medrol®. Three participants provided incomplete information about corticosteroid product names and doses. Thirty-nine of 112 [34.8%] practitioners did not combine viscosupplements with other IA medications or products. Seventeen of 112 [15.2%] participants used viscosupplements with PRP or ACP, 1/112 [0.9%] used antibiotics (product name and dose not specified), 1/112 [0.9%] used ACS, 1/112 [0.9%] used cellular therapy, and 1/112 [0.9%] reported the use of other viscosupplements (Spryng™ and HA). Ten of 112 [8.9%] selected the “other” option to specify how they combine products: viscosupplements with morphine postoperative (*n* = 2), viscosupplements with PRP or triamcinolone (3–6 mg/joint) (*n* = 2), viscosupplements with meloxicam (*n* = 1), viscosupplements with PRP, cellular therapy or other viscosupplements (Arthramid® or Spryng™) (*n* = 1), viscosupplements (Hyvisc® or Evervisc) with PRP or corticosteroids (*n* = 1), viscosupplements with cellular therapy or PRP (*n* = 1), viscosupplements with bupivacaine (*n* = 1), and viscosupplements with dexmedetomidine (*n* = 1). When considering “other” responses that mention viscosupplements with corticosteroids, the total number of participants who combine viscosupplements with corticosteroids increases to 45/112 [40.1%].

Practitioners were asked to mention their typical treatment protocol for viscosupplements. Thirty-six of 112 [32.1%] participants used viscosupplements as a one-time injection, 30/112 [26.8%] participants repeated injection based on long-term clinical response (i.e., ‘maintenance’ therapy performed every 6 months–1 year), 27/112 [24.1%] practitioners repeated injection based on short-term clinical response (i.e., re-injection performed within 3 months of initial therapy), 11/112 [9.8%] practitioners repeated injection every 1–2 weeks for 3 treatments, and 8/112 [7.1%] participants selected the “other” option to specify their personalized protocols. These included: depends on the condition being treated (*n* = 1), sole agent postoperative in tibial plateau level osteotomy (TPLO) that declined PRP (*n* = 1), one time injection and if it is needed once per year (*n* = 1), repeated as needed (*n* = 1), HA in combination with PRP followed by Noltrex®Vet in combination with PRP unless first injection provided good response (*n* = 1), repeated injection based both on short-term and long-term clinical response (*n* = 1), with PRP and repeat in 2 weeks if joint effusion is still present (*n* = 1), following PRP for degenerative joint disease (*n* = 1).

Fifty-one of 112 [45.5%] practitioners mentioned that “some” of their patients seemed to respond after viscosupplement administration, 46/112 [41.1%] of participants mentioned that “a lot” of their cases seemed to respond, 9/112 [8.0%] participants reported that “few” patients seemed to respond, and 6/112 [5.4%] practitioners reported that “all” of their cases seemed to respond to the treatment (Figure 7). There were no differences in perceived proportion of case responders between the use of viscosupplements alone and their association with corticosteroids (*p* = 0.18). Statistical analysis was similarly performed for all other combinations, and no statistically significant differences were observed.

Regarding those positive responders, 59/112 [52.7%] practitioners estimated that their patients had “some” clinical improvement, 48/112 [42.9%] participants reported “substantial” clinical improvement, and 5/112 [4.5%] participants reported “a little” clinical improvement in their cases. None of the practitioners reported “total resolution” (Figure 8). There were no differences in perceived clinical improvement between the use of viscosupplements alone and their association with corticosteroids (*p* = 0.11). Statistical analysis was similarly performed for all other combinations, and no statistically significant differences were observed.

More than half of the participants reported not observing joint flare in their patients. Table 5 summarizes the incidence of acute joint flares observed by practitioners following IA injection with viscosupplements.

### Radionuclides

Survey results for radionuclide therapy are summarized in Table 4. Of the 144 participants that used NSIATs, 24 [16.7%] used radionuclides while 120 [83.3%] did not.

Of the 24 practitioners who used radionuclides, 19 participants [79.2%] had a single specialization or certification, while 5 participants [20.8%] had multiple specializations and/or certifications.

Radionuclide therapy was performed by practitioners whose major disciplinary focus is orthopedic surgery (8/24 [33.3%]) followed by mixed – orthopedic and soft tissue surgery (7/24 [29.2%]), sports medicine and rehabilitation (6/24 [25.0%]), and general practice (3/24 [12.5%]).

Twenty-one of 24 [87.5%] practitioners that used radionuclide had their workplace setting in private practice, and 3/24 [12.5%] in academia.

Most participants that used radionuclide therapy in the USA had their practice located in the northeast (11/24 [45.8%]), followed by the southeast (6/24 [25.0%]), the midwest (6/24 [25.0%]), and the southwest (1/24 [4.2%]). No non-USA residents reported the use of radionuclide therapy.

Due to the small sample size, the previous data was not subjected to statistical analysis.

Practitioners were asked to mention the most common reason they use radionuclide therapy in their patients. Twenty-two of 24 [91.7%] participants mentioned chronic articular pathology needing ‘maintenance’ or routine injections, followed by acute articular pathology (2/24 [8.3%]).

Participants were asked if they administered other IA products simultaneously with radionuclide therapy. All participants (24/24 [100.0%]) reported that they did not combine radionuclide therapy with any other IA medications or products.

Practitioners were asked to mention their typical treatment protocol for radionuclide therapy. Seven of 24 [29.2%] participants used radionuclide therapy once per 12-month interval in elbow joints only, 13/24 [54.2%] participants used radionuclide therapy once per 12-month interval in joints other than the elbow, and 4/24 [16.7%] practitioners selected the “other” option to specify their personalized protocols: once per 12-month interval in any joint (*n* = 3) and once per 12-month interval or longer interval depending on patient clinical assessment at 12 months (*n* = 1).

Thirteen of 24 [54.2%] participants indicated that “a lot” of their cases seemed to respond after radionuclide administration, 5/24 [20.8%] practitioners mentioned that “some” of their patients seemed to respond, 4/24 [16.7%] practitioners reported that “all” of their cases seemed to respond, and 2/24 [8.3%] participants reported that “few” patients seemed to respond to the treatment (Figure 7).

Regarding those positive responders, 14/24 [58.3%] participants estimated that their patients had “substantial” clinical improvement, 7/24 [29.2%] practitioners reported “some” clinical improvement, and 3/24 [12.5%] reported “total resolution” (Figure 8).

Of the total participants, approximately one-third reported an incidence of joint flare in 50% of the dogs treated. Table 5 summarizes the incidence of acute joint flares observed by practitioners following IA injection with radionuclide therapy.

### Other non-steroidal intra-articular therapeutics

Participants were asked to report any additional NSIATs not mentioned in the survey that they routinely use and find helpful in the management of canine OA. Five of 144 [3.5%] practitioners reported the following non-steroidal IA products: meloxicam (*n* = 1), AlphaFlo (*n* = 1), prolozone (*n* = 1), and prolotherapy with dextrose solution (*n* = 2).

## Discussion

This study is the first to gather comprehensive information from canine practitioners on IA injection practices and the first to identify the current clinical use as well as perceived outcomes of NSIATs in canine patients. Regarding the frequency of IA injections, the lack of comparable canine studies and the limited number of responses prevent us from comparing results or asserting that canine IA injections (steroidal and non-steroidal therapies) are commonly performed.

The findings of this study showed that the most injected joint by practitioners was the elbow, followed by the stifle and shoulder. A similar order of frequency was reported in a retrospective study examining the safety of IA injections in canines, which identified the elbow as the most commonly injected joint, followed by the stifle (16). Likewise, a 2024 survey on the use of Polyacrylamide Hydrogel (2.5% iPAAG) in dogs with OA identified the elbow as the most frequently treated joint (17). Reports on the prevalence of elbow joint disease in dogs are limited, but one retrospective study reported a prevalence of 0.56%, identifying OA as the most common cause of elbow joint disease (18). In the authors’ opinion, the prevalence of canine elbow OA, the limited effectiveness of surgical treatment options, and the relative ease of injecting this joint represent plausible hypotheses that may explain why the elbow was reported as the most commonly injected joint. In the case of the stifle, prevalence studies reporting stifle joint disease were not identified, but the prevalence of cranial cruciate ligament disease specifically has been reported to range from 0.56 to 2.55% (19, 20). While the stifle is a relatively easy joint for injection, with a high prevalence of disease, the authors feel it has more effective surgical treatment options when compared to the elbow.

Of the 164 participants who performed IA injections, 144 [87.8%] used NSIATs. Among those who did not use NSIATs, 11 [55.0%] mentioned unclear benefits in the current literature as the primary reason for their choice. Reviews of blood-derived products and stem cell studies reveal significant variability in the quality of published literature and study designs, as well as a lack of description and standardization of regenerative medicine products and techniques (21–24). Reviewers agreed that these inconsistencies and differing outcome measures make it challenging for readers to compare studies and draw conclusions about the therapeutic effects of these therapies (21–24).

Scientific data and articles published regarding the product’s safety and efficacy were the most important criteria influencing participants’ NSIAT selection. For practitioners who used NSIATs and those who did not, the existing evidence in the literature played a crucial role in their decision-making process regarding whether to use NSIATs or which NSIATs to use. This underscores the importance of continued research to investigate the analgesic and disease-modifying effects of NSIATs, to enable clinicians to make well-informed decisions regarding when and how to incorporate these therapies into clinical practice.

The most commonly used and the most commonly preferred NSIATs were PRP, followed by viscosupplements, cellular therapeutics, radionuclide, ACS, and APS. These results differ somewhat from those reported in equine practice, where a survey on the use of NSIATs identified viscosupplements, ACS, and PRP as the most commonly used NSIATs by equine practitioners (15). Another difference noted in the equine survey was that the top reason for using ACS was acute articular pathology, whereas the top reason for using PRP was ligament/tendon pathology. In contrast, in the present canine study, the top reported reason for using both PRP and ACS was chronic articular pathology. The authors speculate that these differences may be partly attributable to variations in survey design and advertising strategies between the two surveys. In particular, the canine survey placed a strong emphasis on IA use, which may have altered the responses provided by the canine practitioner population. Additionally, ACS, more commonly known as IRAP, is not a heavily advertised product in canine medicine as it is in equine medicine. Our survey respondents only reported the use of three different ACS kits, while respondents using PRP mentioned >15 commercial kits utilized for PRP creation. This difference suggests a lower availability of ACS products in the canine market.

Chronic articular pathology needing ‘maintenance’ or routine injections was the primary reason for practitioner use for all NSIATs, except for APS, which was primarily used for acute articular pathology. The increasing prevalence of radiographic and clinical OA in recent years (25, 26), and the fact that most of the available evidence on NSIATs in canines pertains to their application in patients with OA (5, 7, 9, 11, 14, 27–31), may help explain why chronic joint pathology was the leading reason for the use of most NSIATs consulted in this survey. APS research in canine patients has primarily focused on patients with OA (9, 14, 32). Its clinical use in canine patients has also been documented for postoperative IA treatment following cranial cruciate ligament repair, as well as for OA (33). Evidence in the literature regarding the use of APS in canines for the treatment of acute articular pathology was not identified by the authors. One possible reason for its clinical use may stem from equine practice, where acute articular pathology was reported as a common reason for the use of APS by equine practitioners (15).

Most participants [56.9–75.0%] did not combine PRP, ACS, and APS with other IA products. Possible reasons for this include that while studies demonstrate the benefits of PRP when combined with other therapies (34–36), the available evidence shows improvement in both subjective outcomes (6, 29, 37–39) and objective measures (8, 40–42), when used as a sole therapy in the management of naturally occurring OA in different joints. For APS, existing research has examined its efficacy through subjective and objective evaluations as a sole therapy and in comparison to other NSIATs (9, 14, 32), but not in combination with other NSIATs. In the case of ACS, a study in canines demonstrated significant improvement after evaluation with clinical metrology instruments using this product alone and in combination with triamcinolone (4). Although more studies on ACS have been conducted in horses, a recent systematic review concluded that the available evidence in the management of equine OA does not support its effectiveness (43).

Fifty-eight percent of participants who used cellular therapy combined this product with PRP. This overall percentage was calculated based on the number of participant responses reporting the combined use of cellular therapy with PRP, as well as specific combinations of these NSIATs reported under the “other” option for the same question. Certain growth factors are known to regulate the proliferation and differentiation of stem cells (44, 45). This understanding has encouraged the investigation of novel therapies and genetically engineered approaches involving the combination of PRP and cellular products. Recent studies on the combined use of PRP and stem cells in dogs with OA have reported a decrease in various synovial MMPs involved in cartilage degradation (46), as well as synovial pro-inflammatory cytokines such as TNF-*α*, IL-1β, and IL-6 (47). An *in vitro* study suggested that mesenchymal stem cells expressing platelet-derived growth factor might play a protective role in chondrocytes, preventing extracellular matrix degradation (48). *In vivo*, this same study demonstrated an improvement in symptoms, including lameness, weight-bearing, and pain, in an induced OA model. Additionally, a surgically induced canine OA study reported that PRP, alone or in combination with mesenchymal stem cells, positively affected lameness, focal compressive strength, extracellular matrix synthesis, and chondrocyte proliferation compared to controls (36). A few studies on PRP combined with cellular therapy in naturally occurring canine OA models showed improvements in peak vertical force at certain time points (49, 50). However, these studies faced limitations, including the intravenous administration of the combined therapy alongside IA injection, the concurrent use of NSAIDs and other disease-modifying treatments (49), and small sample sizes (49, 50). More research is needed to compare the separate effects of PRP and cell therapy, as well as their combined effect (21, 22). One practitioner mentioned the simultaneous use of cellular therapy and corticosteroids. It is worth noting that *in vitro* exposure of cellular therapy to corticosteroids has been linked to cytotoxic effects in both humans and horses (51, 52).

Eight of the practitioners who reported using NSIATs administered antibiotics intra-articularly in combination with NSIATs. Among them, 4 combined gentamicin with PRP, while 3 used gentamicin simultaneously with cellular therapy. Additionally, one participant combined viscosupplements with an unspecified antibiotic. An *in vitro* study evaluating the effects of various antibiotics on canine chondrocytes and synoviocytes revealed higher cytotoxicity with enrofloxacin and cefazolin compared to other antibiotics (53). The same study also investigated the impact of amikacin, an aminoglycoside class drug, on cell viability. However, to the author’s knowledge, there is a lack of studies evaluating the effects of other aminoglycosides, such as gentamicin, on canine chondrocytes and synoviocytes. In human cartilage, an *in vitro* study demonstrated chondrocyte toxicity with gentamicin, among other antibiotics (54). In equine medicine, aminoglycosides are frequently used as intra-articular antibiotics (55). This last review highlights that all studies examining the effects of aminoglycosides, including gentamicin, have reported varying degrees of joint toxicity in horses. A study assessing the effects of gentamicin and amikacin on cultured equine bone marrow mesenchymal stem cells reported a rapid loss of cell viability, leading to a recommendation against their combined use (56). Based on this and previous *in vitro* findings showing altered cell viability, RNA levels, and gene expression in equine bone marrow mesenchymal stromal cells exposed to antibiotics, including aminoglycosides (57), the authors would advise avoiding the concurrent use of antibiotics and cellular therapy in canines.

Among the participants who used viscosupplements, 45 out of 112 [40.1%] combined this product with corticosteroids. Triamcinolone was the most frequently mentioned corticosteroid, with 30 out of 45 practitioners [66.7%] reporting it. Studies evaluating the efficacy of combining HA with corticosteroids (triamcinolone or methylprednisolone) for treating naturally occurring OA have reported significant improvements in subjective outcome measures (9, 29, 58). However, the use of corticosteroids in canines is controversial due to potential damage to articular cartilage. An *in vitro* pilot study on canine chondral and synovial samples reported a significant loss of cell viability with methylprednisolone, though not with triamcinolone (59). The choice of corticosteroid drug, along with dosage and frequency of corticosteroid injections, are key factors that could positively or negatively influence joint health and should be carefully considered (3, 60–62).

Although the focus of this survey was on NSIATs, it is important to note that, overall, 49/144 practitioners reported the combined use of certain NSIATs with corticosteroids (PRP [*n* = 3]; cellular therapy [*n* = 1], and viscosupplements [*n* = 45]). The authors hypothesize that combining therapies during a single sedation may be influenced by the owner’s financial and time constraints, which may limit their ability to pursue repeated IA injections. Additionally, the authors speculate that concurrent use of a corticosteroid with other therapies may give the clinician the feeling that they are acutely improving joint comfort while awaiting response to the NSIAT of choice. Lastly, the higher percentage use of corticosteroids with a viscosupplement versus other NSIATs may be the result of the commonly held recommendation that HA should be combined when using IA corticosteroids to limit cytotoxic effects. As previously discussed, the combined use of NSIATs and corticosteroids warrants further investigation on cytotoxic effects and overall efficacy of single-agent versus combined outcomes.

Sixty-three of 116 [54.3%] practitioners using PRP and 2/3 [66.7%] practitioners using APS use concurrent oral or systemic injectable NSAIDs. NSAID therapy plays a role in the inhibition of platelet activation and growth factor release (63). This raises concerns about whether NSAIDs might inhibit the effects of PRP and APS. Some practitioners, based on the author’s experience, withhold NSAIDs before and/or after PRP administration. However, the literature does not robustly conclude that NSAIDs are contraindicated with PRP (64). A systematic review assessing human studies on the effect of antiplatelet drugs on growth factor profiles has shown controversial results (63). The same review suggests that the type of PRP activation agent may allow PRP’s therapeutic activity to be maintained despite NSAID or antiplatelet therapy. In animal studies, a canine study demonstrated that carprofen administration did not affect platelet activation or the release of growth factors in canine PRP activated with human gamma thrombin (65). An equine study demonstrated that a single dose of various NSAIDs did not affect the concentrations of growth factors or cytokines in PRP and APS (66). In turn, a recent canine study demonstrated that platelet and leukocyte counts of PRP were not affected after the administration of firocoxib and carprofen (67). Interestingly, this same study demonstrated that both COX-2 selective NSAIDs can affect the production and release of IL-10 from canine platelet-rich gels, suggesting that this should be taken into consideration when using these NSAIDs in conjunction with PRP. Future research in canines on this topic, including NSAIDs and PRP activation agents and the impact of NSAIDs on growth factors and cytokines, is essential to guide clinical decisions.

In the context of PRP, two mechanisms of platelet activation have been described: endogenous and exogenous (23). When asking about activation in the survey, the authors elected to provide the responding clinicians with the sequential options of no activation and physiologic/endogenous activation. This was a purposeful design to determine if respondents realized that endogenous activation will naturally occur upon contact with collagen fibers in the joint environment. Interestingly, 51/116 practitioners reported no activation, while only 30/116 reported physiologic/endogenous activation. This could suggest practitioner confusion over how and where platelet activation occurs with the use of PRP products. This is further highlighted by one respondent who reported activation with citrate dextrose as an “other” option. Citrate dextrose is one of the most commonly used anticoagulants in PRP kits (23), but it is not considered a platelet activation method for the release of growth factors. The debate over whether to activate platelets persists in part because it is argued that endogenous activation may release only a fraction of the growth factors contained within platelets (23). In the human PRP literature, diverse and sometimes conflicting findings regarding activation mechanisms have been reported, without a clear consensus (68). A similar situation exists in equine and canine medicine (69–71). While there was a statistical difference in perceived clinical improvement when comparing “none”/endogenous activation with calcium gluconate and when comparing extracorporeal shockwave with calcium gluconate, the clinical significance of these findings is difficult to interpret due to the subjective nature of the survey. It would be best to rely on experimental studies with direct comparisons of activation methods and objective outcome measurements.

Adipose tissue was the most commonly reported tissue source for cellular therapy among participants, which is consistent with the literature indicating that a large proportion of canine cellular therapy studies have been conducted using this tissue source (22). In the specific case of adipose-derived mesenchymal stem cells, their more frequent use in dogs may be attributed to their ease of accessibility and expansion in culture (22). This contrasts with equine medicine, where cultured bone marrow-derived mesenchymal stem cells represent the predominant culture-expanded cellular therapy used in horses (21). While there was a statistical difference in perceived clinical improvement between adipose and bone marrow sources, the clinical significance of these findings is difficult to interpret due to the subjective nature of the survey. Additionally, the survey did not further differentiate between point-of-care preparations (e.g., bone marrow aspirate concentrate and adipose-derived stromal vascular fraction) and culture-expanded cellular therapies (e.g., bone marrow-derived mesenchymal stem cells and adipose-derived mesenchymal stem cells). Future studies would benefit from exploring practitioners’ preferences regarding tissue source, comparing point-of-care preparations with culture-expanded cellular therapies, and assessing their perceived clinical efficacy.

In the present study, the treatment protocols for PRP, ACS, APS, cellular therapy, and viscosupplements lacked consistency. Product variability, lack of standardization, anecdotal evidence, insufficient high-quality evidence-based studies, off-label usage, and absence of government body approval are factors that may contribute to the inconsistency in the frequency of product administration (3, 21, 22, 72). Although this lack of consistency may affect comparison between studies, the question arises as to whether more than one protocol for the same product can yield a positive outcome. In the case of radionuclide (tin-117m/SynovetinOA®), participants demonstrated significant consistency in the treatment protocol, and none of the participants combined it with another IA product simultaneously, likely due to the standardized protocol required by the manufacturer.

Based on the practitioners’ subjective evaluations, the majority reported a higher perceived proportion of case responders with PRP, ACS, APS, cellular therapy, and radionuclide than with viscosupplements. Notably, over half of the participants using PRP, ACS, cellular therapy, and radionuclide observed “substantial” clinical improvement in their positive responders, suggesting a stronger perceived therapeutic effect. In contrast, while practitioners also reported clinical improvement in their positive responders treated with APS and viscosupplements, the degree of improvement was lower than that reported with the other NSIATs. The lower perceived outcomes with viscosupplements are not surprising to the authors as most viscosupplements are a transient lubricant for the joint in comparison to other NSIATs, which may provide more long-term or substantial joint environment changes. Future studies evaluating different NSIATs concurrently could help provide more impactful comparison testing.

Several survey participants mentioned the use of IA viscosupplements approved for use in other species (e.g., Hyalur®, HY-50®, Hyvisc®, Legend®, Hyalovet®, Hylartin®-V, Synvisc®, Hyonate®). In this survey study, Hyvisc® and Hylartin®-V were the most frequently used by practitioners, both of which are FDA-approved for equine use. The authors speculate that this use of equine products could be related to the lower number of canine viscosupplements products available in the marketplace. It is also the authors’ opinion that off-label use of IA HA products between species is relatively safe, as traditional HA products are derived from rooster combs or microbial fermentation, neither of which is species recipient specific.

Some survey participants mentioned the use of therapeutics not intended for IA injection (e.g., Adequan®, an-bfn 2.2% and meloxicam). Concerning the off-label IA use of Adequan® Canine, the authors consulted the manufacturer, who clarified that the product is intended for intramuscular administration only and that they have not conducted any controlled safety or efficacy studies for IA administration. Similarly, the manufacturer of an-bfn 2.2%, an-vision Inc., was contacted about the off-label use of this intraocular HA product in joints, and they reported that no current safety or efficacy studies have been performed for IA use. Research on the IA use of meloxicam in dogs has been previously performed with a focus on postoperative analgesia; however, no clinically significant response was achieved (73). Further canine studies on the use of IA meloxicam in canine OA treatment could not be identified. While, in general, off-label use in veterinary medicine is a common practice, the use of products not fully evaluated in the IA environment should be avoided until studies are completed, given the unknown risk to the joint and/or a lack of efficacy.

The authors observed several areas of confusion among some practitioners regarding their IA product usage. Based on a few survey responses, the authors believe there may be confusion between ACS, PRP, and APS. Participants were initially asked to mention the products they commonly use in their practice. Two practitioners mentioned the use of ACS, but when specifically asked about this product, they did not respond to these questions. Instead, they answered questions related to PRP or APS, which they had not mentioned as commonly used products. Platelet-rich plasma, ACS, and APS are blood-derived products that differ in their concentration of growth factors and cytokines. In the specific case of APS, the confusion may stem from the fact that it can be considered a variant of ACS (21). For clarity, ACS is a cell-free, serum-based product, in contrast to PRP and APS, which are plasma-based products that contain cells with varying amounts of growth factors and cytokines. The authors also observed confusion among some participants regarding AlphaFlo® product, the PRP processing method, and the donor source for the cellular therapeutic. Two participants classified AlphaFlo®, a canine amniotic tissue allograft, as cellular therapy and derived from an allogeneic donor source. Depending on processing and therapeutic use, amnion can be used intact or as a decellularized matrix and protein dominant product. Intact amnion would be considered a cellular therapeutic as it contains amnion epithelial cells and amnion mesenchymal stromal cells. On the other hand, decellularized amniotic membrane provides an extracellular matrix scaffold for tissue regeneration and repair (74). AlphaFlo® is an acellular tissue allograft rich in extracellular matrix proteins, cytokines, and growth factors. The authors infer that a few practitioners may mistakenly believe the product contains cells and, therefore, have classified it as cellular therapy. Two practitioners reported using the commercial system C-PET Canine Platelet Enhancement Therapy, which prepares PRP through filtration (75), even though filtration was not mentioned as a processing method by the participants. Lastly, a practitioner using DogStem®, identified the donor source as allogeneic. However, DogStem® utilizes a xenogeneic donor source derived from equine umbilical cord (76). The above examples suggest a possible lack of familiarity with the composition and/or action of products that are being used.

While joint flare was reported with all therapeutic agents, the results of this survey suggest that NSIATs do not lead to a high incidence of acute joint flare in a large percentage of treated dogs, In the case of PRP, ACS, APS, cellular therapy and viscosupplements, at least 50% of responders reported joint flare incidence to be 2% or less. This is comparable to what was reported in the similarly performed equine survey study (15). However, it is worth noting that in the case of radionuclide therapy, nearly 30% of participants reported an incidence of acute joint flare in 50% of the treated dogs. Radionuclide therapy in human medicine is known to cause transient or self-limiting radiation induced synovitis in a small number of cases (77). A direct comparison of incidences in humans versus dogs should not be made, as different radioactive agents are used. However, this could suggest why clinicians perceived a higher incidence of joint flare after injection of SynovetinOA®, as some of these cases might represent transient radiation induced synovitis. Although this survey did not assess other complications following IA injection, a recent retrospective study described IA injections in canines as safe, with transient soreness identified as the most common adverse event (16). In this study, bone marrow-derived mesenchymal stem cells were more likely to exhibit transient soreness than other IA agents; however, the study did not include ACS, APS, or radionuclide as retrospectively evaluated IA products (16).

The current study has several limitations. In the absence of a prior survey for comparison and given the lack of knowledge of how many practitioners perform IA injections, the expected number of responses was unknown. A true response rate could not be calculated, as there was no information on how many practitioners viewed the survey link posted on social media. Additionally, it was unknown how many veterinarians with a certification in animal rehabilitation received and responded to the survey, as it was distributed to all certified individuals without the ability to exclude veterinary technicians or physical therapists. For the first survey study on this topic, the authors chose the target audience of boarded or certified surgery and rehabilitation practitioners. Moving forward, future studies could focus on a broader audience or include a new target demographic. Although practitioners were not required to answer questions about products they did not use, the survey’s length may have contributed to some participants abandoning it before completion due to survey fatigue. In addition, some of the survey questions could have been more confusing or complex to answer, which could have resulted in response errors and/or incomplete answers from survey respondents. In this context, some participants who reported the combined use of PRP and cellular therapeutics completed only the questions in the cellular therapeutics section and not those in the PRP section. A similar situation occurred among some participants who reported using both PRP and viscosupplements. The reasons why participants, despite using both products, selected one section and not the other are beyond the scope of this study; however, they may be related to factors such as confusion, survey fatigue, or the selection of the therapeutic option they considered most relevant. The answers to questions about proportion of case responders, clinical improvement, and incidence of joint flare were based on subjective assessment. The subjective assessment has a reliance on self-reported data, which makes their answers susceptible to biases, such as recall bias. Lastly, the survey did not ask participants about the duration of the positive effects of the included NSIATs. This decision was made to minimize recall bias, as accurate recollection of this information could be challenging for practitioners. Information on the duration of effect would be valuable to capture in future studies. Surveys focused on a single NSIAT may allow for more detailed questions, reduce survey fatigue, and provide a more clearly defined target audience.

The results of this survey provide valuable insights into IA injections and the use of non-steroidal joint therapy in canines. As the first survey on this topic in dogs, it showed that most canine practitioners who performed IA injections used NSIATs, with PRP and viscosupplements being the most employed. Safety and efficacy studies on NSIATs are essential to understand their therapeutic response and to better assess the impact of different treatment protocols.

## Data Availability

The raw data supporting the conclusions of this article will be made available by the authors, without undue reservation.

## References

[1] Anderson KL Zulch H O’Neill DG Meeson RL Collins LM. Risk factors for canine osteoarthritis and its predisposing arthropathies: a systematic review. Front Vet Sci. (2020) 7:220. doi: 10.3389/fvets.2020.00220, 32411739 PMC7198754

[2] Johnston SA. Osteoarthritis: joint anatomy, physiology, and pathobiology. Vet Clin North Am Small Anim Pract. (1997) 27:699–723. doi: 10.1016/S0195-5616(97)50076-3, 9243777

[3] Lotsikas PJ. Intra-articular injectates: what to use and why. Vet Clin North Am Small Anim Pract. (2022) 52:967–75. doi: 10.1016/j.cvsm.2022.03.004, 35562212

[4] Alves JC Santos A Jorge P Carreira LM. A comparison of intra-articular blood cell secretome and blood cell secretome with triamcinolone acetonide in dogs with osteoarthritis: a crossover study. Animals. (2022) 12:3358. doi: 10.3390/ani12233358, 36496879 PMC9741238

[5] Black LL Gaynor J Adams C Dhupa S Sams AE Taylor R . Effect of intraarticular injection of autologous adipose-derived mesenchymal stem and regenerative cells on clinical signs of chronic osteoarthritis of the elbow joint in dogs. Vet Ther. (2008) 9:192–200. 19003780

[6] Cuervo B Rubio M Sopena J Dominguez JM Vilar J Morales M . Hip osteoarthritis in dogs: a randomized study using mesenchymal stem cells from adipose tissue and plasma rich in growth factors. Int J Mol Sci. (2014) 15:13437–60. doi: 10.3390/ijms150813437, 25089877 PMC4159804

[7] Donecker J Lattimer JC Gaschen L Aulakh KS. Safety and clinical response following a repeat intraarticular injection of tin-117m (117mSn) colloid in dogs with elbow osteoarthritis. Vet Med Res Rep. (2021) 12:325–35. doi: 10.2147/VMRR.S345144, 34950571 PMC8691448

[8] Fahie MA Ortolano GA Guercio V Schaffer JA Johnston G Au J . A randomized controlled trial of the efficacy of autologous platelet therapy for the treatment of osteoarthritis in dogs. J Am Vet Med Assoc. (2013) 243:1291–7. doi: 10.2460/javma.243.9.1291, 24134578

[9] Franklin SP Franklin AL. Randomized controlled trial comparing autologous protein solution to hyaluronic acid plus triamcinolone for treating hip osteoarthritis in dogs. Front Vet Sci. (2021) 8:713768. doi: 10.3389/fvets.2021.713768, 34395580 PMC8357414

[10] Kim SE Pozzi A Yeh JC Lopez-Velazquez M Yong JAA Townsend S . Intra-articular umbilical cord derived mesenchymal stem cell therapy for chronic elbow osteoarthritis in dogs: a double-blinded, placebo-controlled clinical trial. Front Vet Sci. (2019) 6:474. doi: 10.3389/fvets.2019.00474, 31921927 PMC6932969

[11] Pashuck TD Kuroki K Cook CR Stoker AM Cook JL. Hyaluronic acid versus saline intra-articular injections for amelioration of chronic knee osteoarthritis: a canine model. J Orthop Res. (2016) 34:1772–9. doi: 10.1002/jor.23191, 26867692

[12] Pavarotti GS Hivernaud V Brincin M Roche R Barreau P Festy F . Evaluation of a single intra-articular injection of autologous adipose tissue for the treatment of osteoarthritis: a prospective clinical study in dogs. Vet Comp Orthop Traumatol. (2020) 33:258–66. doi: 10.1055/s-0040-1708524, 32316062

[13] Sawyere DM Lanz OI Dahlgren LA Barry SL Nichols AC Werre SR. Cytokine and growth factor concentrations in canine autologous conditioned serum. Vet Surg. (2016) 45:582–6. doi: 10.1111/vsu.12506, 27357270

[14] Wanstrath AW Hettlich BF Su L Smith A Zekas LJ Allen MJ . Evaluation of a single intra-articular injection of autologous protein solution for treatment of osteoarthritis in a canine population. Vet Surg. (2016) 45:764–74. doi: 10.1111/vsu.12512, 27391909

[15] Velloso Alvarez A Boone LH Braim AP Taintor JS Caldwell F Wright JC . A survey of clinical usage of non-steroidal intra-articular therapeutics by equine practitioners. Front Vet Sci. (2020) 7:579967. doi: 10.3389/fvets.2020.579967, 33195592 PMC7642446

[16] Miller AV Carney PC Markmann A Frye CW. Retrospective analysis describes safety of therapeutic joint injections in dogs. J Am Vet Med Assoc. (2023) 261:397–402. doi: 10.2460/javma.22.11.0483, 36595365

[17] Barnhard JA Tringali AA Caldwell NC Webb KR Levine D Pechette Markley A . Owner-reported outcomes indicate intra-articular 2.5% polyacrylamide hydrogel injection is well tolerated and reduces osteoarthritis signs in dogs. J Am Vet Med Assoc. (2025):1–6. doi: 10.2460/javma.25.06.0398 40997901

[18] O’Neill DG Brodbelt DC Hodge R Church DB Meeson RL. Epidemiology and clinical management of elbow joint disease in dogs under primary veterinary care in the UK. Canine Med Genet. (2020) 7:1–15. doi: 10.1186/s40575-020-0080-5, 32835227 PMC7371807

[19] Witsberger TH Villamil JA Schultz LG Hahn AW Cook JL. Prevalence of and risk factors for hip dysplasia and cranial cruciate ligament deficiency in dogs. J Am Vet Med Assoc. (2008) 232:1818–24. doi: 10.2460/javma.232.12.1818, 18598150

[20] Taylor-Brown FE Meeson RL Brodbelt DC Church DB McGreevy PD Thomson PC . Epidemiology of cranial cruciate ligament disease diagnosis in dogs attending primary-care veterinary practices in England. Vet Surg. (2015) 44:777–83. doi: 10.1111/vsu.12349, 26118493

[21] Bogers SH. Cell-based therapies for joint disease in veterinary medicine: what we have learned and what we need to know. Front Vet Sci. (2018) 5:70. doi: 10.3389/fvets.2018.00070, 29713634 PMC5911772

[22] Brondeel C Pauwelyn G de Bakker E Saunders J Samoy Y Spaas JH. Review: mesenchymal stem cell therapy in canine osteoarthritis research: “Experientia docet” (experience will teach us). Front Vet Sci. (2021) 8:668881. doi: 10.3389/fvets.2021.668881. 34095280 PMC8169969

[23] Carr BJ. Platelet-rich plasma as an orthobiologic: clinically relevant considerations. Vet Clin North Am Small Anim Pract. (2022) 52:977–95. doi: 10.1016/j.cvsm.2022.02.005, 35562219

[24] Carr BJ. Regenerative medicine and rehabilitation therapy in the canine. Vet Clin North Am Small Anim Pract. (2023) 53:801–27. doi: 10.1016/j.cvsm.2023.02.011, 36997410

[25] Enomoto M de Castro N Hash J Thomson A Nakanishi-Hester A Perry E . Prevalence of radiographic appendicular osteoarthritis and associated clinical signs in young dogs. Sci Rep. (2024) 14:2827. doi: 10.1038/s41598-024-52324-9, 38310147 PMC10838335

[26] Wright A Amodie DM Cernicchiaro N Lascelles BDX Pavlock AM Roberts C . Identification of canine osteoarthritis using an owner-reported questionnaire and treatment monitoring using functional mobility tests. J Small Anim Pract. (2022) 63:609–18. doi: 10.1111/jsap.13500, 35385129 PMC9543207

[27] Botto R Riccio V Galosi L Rossi G Vincenzetti S Tambella AM . Effects of intra-articular autologous adipose micrograft for the treatment of osteoarthritis in dogs: a prospective, randomized, controlled study. Animals. (2022) 12:1844. doi: 10.3390/ani12141844, 35883392 PMC9311928

[28] Cuervo B Rubio M Chicharro D Damia E Santana A Carrillo JM . Objective comparison between platelet rich plasma alone and in combination with physical therapy in dogs with osteoarthritis caused by hip dysplasia. Animals. (2020) 10:175. doi: 10.3390/ani10020175, 31972961 PMC7070503

[29] Franklin SP Cook JL. Prospective trial of autologous conditioned plasma versus hyaluronan plus corticosteroid for elbow osteoarthritis in dogs. Can Vet J. (2013) 54:881–4. 24155495 PMC3743576

[30] Kim S Elam L Johnson V Hess A Webb T Dow S . Intra-articular injections of allogeneic mesenchymal stromal cells vs. high molecular weight hyaluronic acid in dogs with osteoarthritis: exploratory data from a double-blind, randomized, prospective clinical trial. Front Vet Sci. (2022) 9:890704. doi: 10.3389/fvets.2022.890704, 35747237 PMC9209755

[31] Vilar JM Batista M Morales M Santana A Cuervo B Rubio M . Assessment of the effect of intraarticular injection of autologous adipose-derived mesenchymal stem cells in osteoarthritic dogs using a double blinded force platform analysis. BMC Vet Res. (2014) 10:143. doi: 10.1186/1746-6148-10-143, 24984756 PMC4085658

[32] Franklin SP. A pilot clinical study assessing treatment of canine hip dysplasia using autologous protein solution. Front Vet Sci. (2019) 6:243. doi: 10.3389/fvets.2019.00243, 31448294 PMC6696975

[33] King W Cawood K Bookmiller M. The use of autologous protein solution (pro-stride®) and leukocyte-rich platelet-rich plasma (Restigen®) in canine medicine. Vet Med Res Rep. (2021) 12:53–65. doi: 10.2147/VMRR.S286913, 33777723 PMC7989049

[34] Lee M Kim J Kwak H Woo H Han J Yayon A . A placebo-controlled study comparing the efficacy of intra-articular injections of hyaluronic acid and a novel hyaluronic acid-platelet-rich plasma conjugate in a canine model of osteoarthritis. J Orthop Surg Res. (2019) 14:314. doi: 10.1186/s13018-019-1352-1, 31533754 PMC6749694

[35] Sanghani-Kerai A Black C Cheng S Collins L Schneider N Blunn G . Clinical outcomes following intra-articular injection of autologous adipose- derived mesenchymal stem cells for the treatment of osteoarthritis in dogs characterized by weight-bearing asymmetry. Bone Joint Res. (2021) 10:650–8. doi: 10.1302/2046-3758.1010.BJR-2020-0540.R1, 34628940 PMC8559970

[36] Yun S Ku SK Kwon YS. Adipose-derived mesenchymal stem cells and platelet-rich plasma synergistically ameliorate the surgical-induced osteoarthritis in beagle dogs. J Orthop Surg Res. (2016) 11:9. doi: 10.1186/s13018-016-0342-9, 26768536 PMC4714505

[37] Alves JC Santos A Jorge P Lavrador C Carreira LM. A report on the use of a single intra-articular administration of autologous platelet therapy in a naturally occurring canine osteoarthritis model-a preliminary study. BMC Musculoskelet Disord. (2020) 21:127. doi: 10.1186/s12891-020-3140-9, 32106842 PMC7047415

[38] Alves JC Santos A Jorge P. Platelet-rich plasma therapy in dogs with bilateral hip osteoarthritis. BMC Vet Res. (2021) 17:207. doi: 10.1186/s12917-021-02913-x, 34090433 PMC8180029

[39] Aminkov KB Mehandzhiyski NH Aminkov BY Zlateva-Panayotova NZ. Application of platelet-rich plasma for canine osteoarthritis treatment - a clinical series. Bulg J Vet Med. (2021) 24:601–7. doi: 10.15547/bjvm.2019-0095

[40] Silva RF Carmona JU Rezende CMF. Intra-articular injections of autologous platelet concentrates in dogs with surgical reparation of cranial cruciate ligament rupture: a pilot study. Vet Comp Orthop Traumatol. (2013) 26:285–90. doi: 10.3415/VCOT-12-06-0075, 23612687

[41] Venator KP Frye CW Gamble LJ Wakshlag JJ. Assessment of a single intra-articular stifle injection of pure platelet rich plasma on symmetry indices in dogs with unilateral or bilateral stifle osteoarthritis from long-term medically managed cranial cruciate ligament disease. Vet Med Res Rep. (2020) 11:31–8. doi: 10.2147/VMRR.S238598, 32215259 PMC7082539

[42] Vilar J Manera M Santana A Spinella G Rodriguez O Rubio M . Effect of leukocyte-reduced platelet-rich plasma on osteoarthritis caused by cranial cruciate ligament rupture: a canine gait analysis model. PLoS One. (2018) 13:e0194752. doi: 10.1371/journal.pone.0194752, 29554150 PMC5858837

[43] Della Tommasa S Brehm W Farì G Bernetti A Imperante A. Use of autologous conditioned serum (ACS) for osteoarthritis treatment in horses: a systematic review of clinical data. Vet Sci. (2023) 10:707. doi: 10.3390/vetsci10120707, 38133258 PMC10747612

[44] Lai F Kakudo N Morimoto N Taketani S Hara T Ogawa T . Platelet-rich plasma enhances the proliferation of human adipose stem cells through multiple signaling pathways. Stem Cell Res Ther. (2018) 9:107. doi: 10.1186/s13287-018-0851-z, 29661222 PMC5902971

[45] Tobita M Tajima S Mizuno H. Adipose tissue-derived mesenchymal stem cells and platelet-rich plasma: stem cell transplantation methods that enhance stemness. Stem Cell Res Ther. (2015) 6:215. doi: 10.1186/s13287-015-0217-8, 26541973 PMC4635588

[46] Arican M Uney K Parlak K Uzunlu EO Sonmez G. Proteases and collagenase enzymes activity after autologous platelet- rich plasma, bio-physically activated PRP and stem cells for the treatment of osteoarthritis in dogs. Kafkas Univ Vet Fak Derg. (2022) 28:437–45. doi: 10.9775/kvfd.2022.27357

[47] Parlak K Uney K Uzunlu EO Yalcin M Arican M. The effect of intra-articular platelet-rich plasma, bio-physically activated PRP and mesenchymal stem cell administration for interleukins in dogs with osteoarthritis. Vet Arh. (2022) 92:459–68. doi: 10.24099/vet.arhiv.1695

[48] Oh J Son Y Kim W Kwon O Kang B. Mesenchymal stem cells genetically engineered to express platelet-derived growth factor and heme oxygenase-1 ameliorate osteoarthritis in a canine model. J Orthop Surg Res. (2021) 16:43. doi: 10.1186/s13018-020-02178-4, 33430899 PMC7802278

[49] Upchurch DA Renberg WC Roush JK Milliken GA Weiss ML. Effects of administration of adipose-derived stromal vascular fraction and platelet-rich plasma to dogs with osteoarthritis of the hip joints. Am J Vet Res. (2016) 77:940–51. doi: 10.2460/ajvr.77.9.940, 27580105

[50] Vilar JM Morales M Santana A Spinella G Ramon M Cuervo B . Controlled, blinded force platform analysis of the effect of intraarticular injection of autologous adipose-derived mesenchymal stem cells associated to PRGF-Endoret in osteoarthritic dogs. BMC Vet Res. (2013) 9:131. doi: 10.1186/1746-6148-9-131 23819757 PMC3716942

[51] Edmonds RE Garvican ER Smith RKW Dudhia J. Influence of commonly used pharmaceutical agents on equine bone marrow-derived mesenchymal stem cell viability. Equine Vet J. (2017) 49:352–7. doi: 10.1111/evj.12590, 27160051

[52] Wyles CC Houdek MT Wyles SP Wagner ER Behfar A Sierra RJ. Differential cytotoxicity of corticosteroids on human mesenchymal stem cells. Clin Orthop Relat Res. (2015) 473:1155–64. doi: 10.1007/s11999-014-3925-y, 25187334 PMC4317436

[53] Newman RJ Chow L Goodrich LR Lambrechts NE Dow SW Pezzanite LM. Susceptibility of canine chondrocytes and synoviocytes to antibiotic cytotoxicity in vitro. Vet Surg. (2021) 50:650–8. doi: 10.1111/vsu.13591, 33606293

[54] Mayr HO Regenbrecht N Mayr MF Riedel B Hart ML Schmal H . Effect of vancomycin, gentamicin and clindamycin on cartilage cells in vitro. Biomedicine. (2023) 11:3143. doi: 10.3390/biomedicines11123143, 38137364 PMC10740484

[55] Pezzanite LM Hendrickson DA Dow S Stoneback J Chow L Krause D . Intra-articular administration of antibiotics in horses: justifications, risks, reconsideration of use and outcomes. Equine Vet J. (2022) 54:24–38. doi: 10.1111/evj.13502, 34459027

[56] Bohannon LK Owens SD Walker NJ Carrade DD Galuppo LD Borjesson DL. The effects of therapeutic concentrations of gentamicin, amikacin and hyaluronic acid on cultured bone marrow-derived equine mesenchymal stem cells. Equine Vet J. (2013) 45:732–6. doi: 10.1111/evj.12045, 23448189

[57] Parker RA Clegg PD Taylor SE. The in vitro effects of antibiotics on cell viability and gene expression of equine bone marrow-derived mesenchymal stromal cells. Equine Vet J. (2012) 44:355–60. doi: 10.1111/j.2042-3306.2011.00437.x, 21883415

[58] Alves JC Santos A Jorge P Lavrador C Carreira LM. A pilot study on the efficacy of a single intra-articular administration of triamcinolone acetonide, hyaluronan, and a combination of both for clinical management of osteoarthritis in police working dogs. Front Vet Sci. (2020) 7:512523. doi: 10.3389/fvets.2020.512523., 33282924 PMC7690322

[59] Sherman SL Khazai RS James CH Stoker AM Flood DL Cook JL. In vitro toxicity of local anesthetics and corticosteroids on chondrocyte and synoviocyte viability and metabolism. Cartilage. (2015) 6:233–40. doi: 10.1177/1947603515594453, 26425261 PMC4568732

[60] Bensa A Salerno M Boffa A de Girolamo L Laver L Magalon J . Corticosteroid injections for the treatment of osteoarthritis present a wide spectrum of effects ranging from detrimental to disease-modifying: a systematic review of preclinical evidence by the ESSKA Orthobiologic initiative. Knee Surg Sports Traumatol Arthrosc. (2024) 32:2725–45. doi: 10.1002/ksa.12242, 38813889

[61] Pirri C Sorbino A Manocchio N Pirri N Devito A Foti C . Chondrotoxicity of intra-articular injection treatment: a scoping review. Int J Mol Sci. (2024) 25:7010. doi: 10.3390/ijms25137010, 39000119 PMC11241418

[62] Vandeweerd JM Zhao Y Nisolle JF Zhang W Zhihong L Clegg P . Effect of corticosteroids on articular cartilage: have animal studies said everything? Fundam Clin Pharmacol. (2015) 29:427–38. doi: 10.1111/fcp.12137, 26211421

[63] Frey C Yeh PC Jayaram P. Effects of antiplatelet and nonsteroidal anti-inflammatory medications on platelet-rich plasma: a systematic review. Orthop J Sports Med. (2020) 8:2325967120912841. doi: 10.1177/2325967120912841, 32426401 PMC7218995

[64] Magruder M Rodeo SA. Is antiplatelet therapy contraindicated after platelet-rich plasma treatment? A narrative review. Orthop J Sports Med. (2021) 9:23259671211010510. doi: 10.1177/23259671211010510, 34179207 PMC8202276

[65] Ludwig HC Birdwhistell KE Brainard BM Franklin SP. Use of a cyclooxygenase-2 inhibitor does not inhibit platelet activation or growth factor release from platelet-rich plasma. Am J Sports Med. (2017) 45:3351–7. doi: 10.1177/0363546517730578, 28952781

[66] Brown KA Gregorio EN Barot D Usimaki A Linardi RL Missanelli JR . Single-dose nonsteroidal anti-inflammatory drugs in horses have no impact on concentrations of cytokines or growth factors in autologous protein solution and platelet-rich plasma. Am J Vet Res. (2024) 85:23.11.0258. doi: 10.2460/ajvr.23.11.0258, 38346393

[67] Ospina J Carmona JU López C. Short-term effects of two COX-2 selective non-steroidal anti-inflammatory drugs on the release of growth factors and cytokines from canine platelet-rich gel supernatants. Gels. (2024) 10:396. doi: 10.3390/gels10060396, 38920942 PMC11202787

[68] Pachito DV Bagattini ÂM de Almeida AM Mendrone-Júnior A Riera R. Technical procedures for preparation and administration of platelet-rich plasma and related products: a scoping review. Front Cell Dev Biol. (2020) 8:598816. doi: 10.3389/fcell.2020.598816, 33363154 PMC7759516

[69] Textor JA Tablin F. Activation of equine platelet-rich plasma: comparison of methods and characterization of equine autologous thrombin. Vet Surg. (2012) 41:784–94. doi: 10.1111/j.1532-950X.2012.01016.x, 22742830

[70] Seabaugh KA Thoresen M Giguère S. Extracorporeal shockwave therapy increases growth factor release from equine platelet-rich plasma *in vitro*. Front Vet Sci. (2017) 4:205. doi: 10.3389/fvets.2017.00205, 29270410 PMC5726030

[71] Franklin SP Birdwhistell KE Strelchik A Garner BC Brainard BM. Influence of cellular composition and exogenous activation on growth factor and cytokine concentrations in canine platelet-rich plasmas. Front Vet Sci. (2017) 4:40. doi: 10.3389/fvets.2017.00040, 28424777 PMC5380674

[72] Carr BJ Miller AV Colbath AC Peralta S Frye CW. Literature review details and supports the application of platelet-rich plasma products in canine medicine, particularly as an orthobiologic agent for osteoarthritis. J Am Vet Med Assoc. (2024) 262:S8–S15. doi: 10.2460/javma.23.12.0692 38382202

[73] Moak P Hosgood G Rowe E Lemke KA. Evaluation of intra-articular and subcutaneous administration of meloxicam for postoperative analgesia following stifle surgery in dogs. Vet Comp Orthop Traumatol. (2011) 24:32–8. doi: 10.3415/VCOT-10-04-0059, 21103650

[74] Ingraldi AL Audet RG Tabor AJ. The preparation and clinical efficacy of amnion-derived membranes: a review. J Funct Biomater. (2023) 14:531. doi: 10.3390/jfb14100531, 37888195 PMC10607219

[75] Carr BJ Canapp SO Mason DR Cox C Hess T. Canine platelet-rich plasma systems: a prospective analysis. Front Vet Sci. (2016) 2:73. doi: 10.3389/fvets.2015.00073, 26779493 PMC4700921

[76] Punzon E Salguero R Totusaus X Mesa-Sanchez C Badiella L Garcia-Castillo M . Equine umbilical cord mesenchymal stem cells demonstrate safety and efficacy in the treatment of canine osteoarthritis: a randomized placebo-controlled trial. J Am Vet Med Assoc. (2022) 260:1947–55. doi: 10.2460/javma.22.06.0237, 36198051

[77] Kampen WU Boddenberg-Pätzold B Fischer M Gabriel M Klett R Konijnenberg M . The EANM guideline for radiosynoviorthesis. Eur J Nucl Med Mol Imaging. (2022) 49:681–708. doi: 10.1007/s00259-021-05541-7, 34671820 PMC8803784

