# Supplementary material for: A survey of the clinical usage of intra-articular therapeutics in dogs by veterinary practitioners with a focus on non-steroidal therapies
Source: Front Vet Sci. 2026 Feb 11;13:1761681. doi: 10.3389/fvets.2026.1761681 (PMC12932153; doi:10.3389/fvets.2026.1761681)
Supplement: Supplementary file 1 [file Supplementary_file_1.docx]

Supplementary Item 1
 A Survey of the Clinical Usage of Non-Steroidal Intra-Articular Therapeutics in Dogs by Veterinary Practitioners - Questionnaire

Clinical usage of non-steroidal intra-articular therapeutics (NSIATs) in dogs, including biologic (e.g., platelet-rich plasma, autologous conditioned serum, autologous protein solution, and cellular therapies) and synthetic products (e.g., polyacrylamide gel, hyaluronic acid, and radionuclide) have increased significantly within recent years. There is limited evidence in the literature regarding clinical usage, injection frequency, perceived outcomes, and clinical reasoning for product usage. This questionnaire is being distributed to veterinarians with one or more of the following certifications: diplomate of the American College of Veterinary Surgeons (ACVS), diplomate of the American College of Veterinary Sports Medicine and Rehabilitation (ACVSMR), diplomate of the European College of Veterinary Surgeons (ECVS), diplomate of the European College of Veterinary Sports Medicine and Rehabilitation (ECVSMR), Certified Canine Rehabilitation Therapist (CCRT), Certified Canine Rehabilitation Practitioner (CCRP), Certified Canine Rehabilitation Veterinarian (CCRV), Certified Companion Animal Rehabilitation Therapist (CCAT) and/or any other rehabilitation certification not mentioned above to better understand these clinical questions. Information gathered from the survey will be disseminated through presentations and publication. 

The survey will take approximately 15-20 minutes to complete. Taking the survey is voluntary. The participant may exit the survey at any time should they decide they no longer wish to participate.

We appreciate your time and participation in the survey.

If you have any questions, comments, or concerns please feel free to contact the principal investigator Dr. Camila Sepulveda at [czs0168@auburn.edu](mailto:czs0168@auburn.edu) or the faculty advisor principal investigator Dr. Kayla Corriveau at [kmc0118@auburn.edu](mailto:kmc0118@auburn.edu)

Sincerely,

Kayla Corriveau, DVM, DACVS-SA

Camila Sepulveda, DVM, CCRP

*****Line represents new slide*****

**Demographics:**

1. Are you a licensed veterinarian?
   1. Yes
   2. No

*****If “No” was answered, the survey finishes, and the response is not recorded in the final results as the participant did not meet inclusion criteria**.***

1. When did you obtain your veterinary license? Please specify year _____________
2. Which of the following diplomat and/or certification status apply to you? Check all that apply and please indicate the year your diplomat and/or certification was obtained.
   1. American College of Veterinary Surgeons (ACVS). Year obtained_______
   2. American College of Veterinary Surgeons – Small Animal (ACVS-SA). Year obtained_______
   3. American College of Veterinary Surgeons – Large Animal (ACVS-LA) (with clinical exposure to dogs). Year obtained _______
   4. American College of Veterinary Sports Medicine & Rehabilitation (ACVSMR) – Canine. Year obtained _______
   5. American College of Veterinary Sports Medicine & Rehabilitation (ACVSMR) – Equine (with clinical exposure to dogs). Year obtained _______
   6. European College of Veterinary Surgeons (ECVS) – Small Animal. Year obtained_______
   7. European College of Veterinary Surgeons (ECVS) – Large Animal (with clinical exposure to dogs). Year obtained_______
   8. European College of Veterinary Sports Medicine & Rehabilitation (ECVSMR) – Small Animal. Year obtained_______
   9. European College of Veterinary Sports Medicine & Rehabilitation (ECVSMR) – Large Animal (with clinical exposure to dogs). Year obtained_______
   10. Certified Canine Rehabilitation Therapist (CCRT). Year obtained _______
   11. Certified Canine Rehabilitation Practitioner (CCRP). Year obtained _______
   12. Certified Canine Rehabilitation Veterinarian (CCRV). Year obtained _______
   13. Certified Companion Animal Rehabilitation Therapist (CCAT). Year obtained _______
   14. Other rehabilitation certification. Please enter certification abbreviation and year obtained_______
3. Of your **total patient caseload**, which statement best describes your canine caseload?
   1. 0%-25%
   2. 26%-50%
   3. 51%-75%
   4. 76%-100%
4. What is your major disciplinary focus?
   1. Sports medicine and rehabilitation
   2. Orthopedic surgery
   3. Soft tissue surgery
   4. Mixed - orthopedic and soft tissue surgery
   5. General practice
   6. Other - Please specify _____________
5. Of your **canine caseload**, which statement best describes your canine orthopedic percentile?
   1. 0%-25% Orthopedics
   2. 26%-50% Orthopedics
   3. 51%-75% Orthopedics
   4. 76%-100% Orthopedics
6. What is your workplace setting?
   1. Academia
   2. Private Practice
   3. Mobile Practice

**Geographic location:**


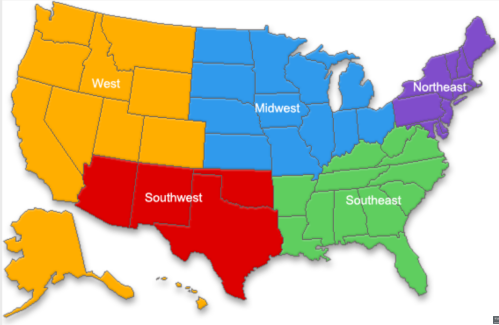


1. Where is your primary practice located?
   1. Southeast United States
   2. Northeast United States
   3. Midwest United States
   4. Southwest United States
   5. West United States
   6. Outside the United States - Please specify the country in which you practice_____________

**Injection Frequency:**

1. On average, how many dogs would you estimate that you performed any kind of joint injections in the **last three months**? This includes **BOTH** steroidal and non-steroidal intra-articular therapies.
   1. None
   2. Less than 3 dogs
   3. 3 to 5 dogs
   4. 6 to 10 dogs
   5. 11 to 15 dogs
   6. 16 to 20 dogs
   7. 21 dogs or more
2. Please rank from the most common joint to the least common joint that you use intra-articular injections on. Click bubbles in order from the most common (1) to the least common (2-7). Leave the joints that you do **NOT** inject blank.
   1. Shoulder
   2. Elbow
   3. Carpus
   4. Hip
   5. Stifle
   6. Tarsus
   7. Other - Please specify _____________
3. Do you use **non-steroidal** intra-articular therapeutics (e.g., platelet-rich plasma, autologous conditioned serum, autologous protein solution, cellular therapeutics, hyaluronic acid, polyacrylamide gel, and radionuclide) in your practice? Even if you do not use these products, your opinion is important.
   1. Yes
   2. No

*****If no, the survey finishes after question number 4 below**.***

*****If yes, the survey continues on to the next section**.***

1. Rank at least the top 2 reasons from most influential (1) to least influential (2-8) to your reasoning for not using non-steroidal intra-articular therapeutics in your practice.
   1. Unclear benefits in current literature
   2. Lack of training
   3. Lack of personal clinical experience
   4. Cost concerns of the product or instrumentation
   5. Lack of product availability
   6. Lack of client interest
   7. Other # 1 – Please specify _____________
   8. Other # 2 - Please specify _____________

**Overall Usage of Non-Steroidal Intra-Articular Therapeutics:**

1. On average, how many dogs have you injected with **non-steroidal** intra-articular therapeutics in the **last 12 months**?
   1. Less than 5 dogs
   2. 5 to 9 dogs
   3. 10 to 19 dogs
   4. 20 to 49 dogs
   5. 50 dogs or more
2. Please rank the **intra-articular products that you use most commonly in your practice** to treat canine joint related pathology from most used (1) to least used (2-8). Leave the products that you do not use blank.
   1. Corticosteroids (methylprednisolone acetate - Depomedrol™, triamcinolone acetonide - Kenalog™, dexamethasone sodium phosphate - Decadron phosphate™, betamethasone - Betaject™)
   2. Viscosupplements (hyaluronic acid, hyaluronate, polyacrylamide hydrogel, Polyglycan™, Arthramid® Vet, Synamid®, Noltrex®, Legend®, Hyvisc®, Synvisc®, Hyalovet®, Hylartin V®, Spryng™)
   3. Platelet-rich plasma (PRP)
   4. Autologous conditioned serum (interleukin-1-receptor antagonist - IRAP products, Orthokine®)
   5. Autologous protein solution (i.e., Pro-Stride™)
   6. Cellular therapy (stem/mesenchymal/stromal cells cultured or concentrated, tissue particulates)
   7. Radionuclides (tin-117m - Synovetin OA™)
   8. Other - Please specify _____________
3. Please rank at least the top 3 reasons from most influential (1) to least influential (2-8) to your decision as to which intra-articular therapy you preferentially use.
   1. Scientific data and articles published regarding the product’s safety and efficacy
   2. Personal experience with the product
   3. Availability of the product
   4. The specific joint being treated
   5. Cost of product
   6. Specific condition being treated
   7. Client request
   8. Other - Please specify _____________
4. Please rank the following **non-steroidal** intra-articular therapeutics in order of **your preference** **(regardless of client preference or product availability)** when treating joint related conditions in dogs. Click bubbles in order from most preferred (1) to least (2-7) preferred products. Leave the products that you do not use blank.
   1. Viscosupplements (hyaluronic acid, hyaluronate, polyacrylamide hydrogel, Polyglycan™, Arthramid® Vet, Synamid®, Noltrex®, Legend®, Hyvisc®, Synvisc®, Hyalovet®, Hylartin V®, Spryng™)
   2. Platelet-rich plasma (PRP)
   3. Autologous conditioned serum (interleukin-1-receptor antagonist - IRAP products, Orthokine®)
   4. Autologous protein solution (i.e., Pro-Stride™)
   5. Cellular Therapy (stem/mesenchymal/stromal cells cultured or concentrated, tissue particulates)
   6. Radionuclides (tin-117m - Synovetin OA™)
   7. Other - Please specify _____________

**Platelet-Rich Plasma (PRP)**

Platelet-rich plasma (PRP) is a product obtained from the dog’s own blood. The blood is filtered or centrifuged to obtain plasma with an increased number of platelets rich in growth factors. A specific method of processing PRP is known as autologous conditioned plasma (ACP).

1. Do you use platelet-rich plasma (PRP) or autologous conditioned plasma (ACP) to treat your patients? If yes, more questions will follow.
   1. Yes
   2. No

*****If no, the survey jumps to the next topic area**.***

*****If yes, the survey continues on**.***

1. How is the platelet-rich plasma (PRP) or autologous conditioned plasma (ACP) processed in your practice prior to administration?
   1. Manual centrifugation
   2. Centrifugation using a commercialized kit and centrifuge
   3. Filtration
   4. Outside laboratory or referral center
   5. It is not processed. It is a freeze-dried product reconstituted with sterile water (i.e., PrecisePRP^TM^).
   6. Other - Please specify _________
2. If you use a commercialized kit to process platelet-rich plasma (PRP) or autologous conditioned plasma (ACP), what kit(s) do you use? Select all that apply.
   1. Arthrex Angel cPRP®, Arthrex Orthobiologics
   2. Arthrex ACP ® Double-syringe system, Arthrex Orthobiologics
   3. Arthrex Max™ Platelet Rich Plasma, Arthrex Orthobiologics
   4. CRT Pure PRP®, Companion Regenerative Therapies
   5. C-PET Canine Platelet Enhancement Therapy, Pall Corporation
   6. MediVet PRP, MediVet America/PureVet PRP, Ardent Animal Health
   7. ProTec PRP, Pulse Veterinary Technologies
   8. Restigen ® PRP, Zoetis
   9. SmartPReP ® ACP+ System, Harvest Technologies
   10. Stryker RegenKit ®, Regen Lab USA
   11. TropoVet^TM^ PRP, Estar Medical
   12. Other - Please specify _____________
   13. None
3. What activation agent do you use with platelet-rich plasma (PRP) or autologous conditioned plasma (ACP) administration?
   1. None
   2. Physiologic stimulation (aka endogenous activation) (allow the joint environment to activate the platelets)
   3. Calcium chloride
   4. Calcium gluconate
   5. Bovine thrombin
   6. Human thrombin (HGT)
   7. Freeze/thaw cycles
   8. Extracorporeal shockwave
   9. Other - Please specify: _________
4. Do you administer concurrent oral or systemic injectable non-steroidal anti-inflammatory medication when administering intra-articular platelet-rich plasma (PRP) or autologous conditioned plasma (ACP)?
   1. Yes
   2. No
5. Do you ensure that the dog is **not** on any specific medications prior to pulling and processing the platelet-rich plasma (PRP) or autologous conditioned plasma (ACP)?
   1. No
   2. Yes – Please specify which drugs _____________
6. Please rank at least the top 2 most common reasons you use platelet-rich plasma (PRP) or autologous conditioned plasma (ACP) in your patients. Click bubbles in order from most important (1) to least (2-7) important reasons.
   1. Preventative or prophylactic measure
   2. Acute articular pathology
   3. Chronic articular pathology needing ‘maintenance’ or routine injections
   4. Post-operative therapy
   5. Ligament or tendon lesions
   6. Tendon sheath or bursa applications
   7. Other - Please specify _____________
7. When using platelet-rich plasma (PRP) or autologous conditioned plasma (ACP), what other intra-articular products do you administer simultaneously? If you use more than two products simultaneously, please list the details in the ‘other’ selection.
   1. None
   2. Antibiotic - Please specify product name and dose (mg/kg) ____________
   3. Autologous conditioned serum (IRAP)
   4. Autologous protein solution (i.e., Pro-Stride ™)
   5. Cellular therapeutics (stem/stromal/progenitor cell therapy cultured or concentrated tissue particles)
   6. Corticosteroids - Please specify product name and dose (mg/kg) _________
   7. Viscosupplement (hyaluronic acid, polyacrylamide hydrogel, collagen-elastin, glucosamine, chondroitin)
   8. Radionuclide therapy (Synovetin OA®)
   9. Other - Please specify _____________
8. If you use platelet-rich plasma (PRP) or autologous conditioned plasma (ACP) for intra-articular therapy, what would your typical treatment protocol be?
   1. One time injection
   2. Repeat injection every 1-2 weeks for 3 treatments
   3. Repeat injection based on short-term clinical response (i.e., re-injection performed within 3 months of initial therapy)
   4. Repeat injection based on long-term clinical response (i.e., ‘maintenance’ therapy performed every 6 mo - 1 yr)
   5. Other - Please specify _____________
9. In your population of dogs receiving platelet-rich plasma (PRP) or autologous conditioned plasma (ACP) intra-articularly, how many cases seem to respond?
   1. Few
   2. Some
   3. A lot
   4. All of them
10. In those positive responders, how much clinical improvement do you tend to see?
    1. A little
    2. Some
    3. Substantial
    4. Total resolution
11. When administering platelet-rich plasma (PRP) or autologous conditioned plasma (ACP) intra-articularly, what incidence of acute joint flares have you encountered post-administration? A joint flare is defined as a period of increased disease activity or worsening of clinical signs in a particular joint (e.g., increased joint pain, inflammation, and lameness).
    1. None
    2. 1 in 50 dogs (2%)
    3. 1 in 20 dogs (5%)
    4. 1 in 10 dogs (10%)
    5. 1 in 5 dogs (20%)
    6. 1 in 2 dogs (50%)
    7. All cases

**Autologous Conditioned Serum (IRAP)**

Autologous conditioned serum, also known as IRAP, is obtained from the dogs’ blood following collection into specialized syringes (containing treated glass beads) and whole blood incubation. The serum is then collected and administered, or aliquots are frozen for subsequent injection. Autologous conditioned serum is enriched in growth factors and anti-inflammatory cytokines like the interleukin-1 receptor antagonist (IL-1Ra). Autologous conditioned serum systems for intra-articular injection include the following:

- Orthokine ® Vet IRAP 10, Dechra
- IRAP II ™ Systems, Arthrex Orthobiologics
- IRAP ProEAS ™ Systems, Arthrex Orthobiologics
- MediVet ACS, MediVet America

1. Do you use autologous conditioned serum (IRAP) to treat your patients? If yes, more questions will follow.
   1. Yes
   2. No

*****If no, the survey jumps to the next topic area**.***

*****If yes, the survey continues on**.***

1. What autologous conditioned serum (IRAP) commercial kit do you use?
   1. Orthokine ® Vet IRAP 10, Dechra
   2. IRAP II ™ Systems, Arthrex Orthobiologics
   3. IRAP ProEAS ™ Systems, Arthrex Orthobiologics
   4. MediVet ACS, MediVet America
   5. Other - Please specify _____________
2. Please rank at least the top 2 most common reasons you use autologous conditioned serum (IRAP) in your patients. Click bubbles in order from most important (1) to least (2-7) important reason.
   1. Preventative or prophylactic measure
   2. Acute articular pathology
   3. Chronic articular pathology needing ‘maintenance’ or routine injections
   4. Post-operative therapy
   5. Ligament or tendon lesions
   6. Tendon sheath or bursa applications
   7. Other - Please specify _____________
3. When using autologous conditioned serum (IRAP) intra-articularly, what other additional intra-articular products do you administer simultaneously? If you use more than two products simultaneously, please list the details in the ‘other’ selection.
   1. None
   2. Antibiotic - Please specify product name and dose (mg/kg) _____________
   3. Platelet-rich plasma (PRP) or autologous conditioned plasma (ACP)
   4. Autologous protein solution (i.e., Pro-Stride ™)
   5. Cellular therapeutics (stem/stromal/progenitor cell therapy cultured or concentrated tissue particles)
   6. Corticosteroid - Please specify product name and dose (mg/kg) _____________
   7. Viscosupplement (hyaluronic acid, polyacrylamide hydrogel, collagen-elastin, glucosamine, chondroitin)
   8. Radionuclide therapy (Synovetin OA®)
   9. Other - Please specify _____________
4. If you use autologous conditioned serum (IRAP) intra-articularly, what would your typical treatment protocol be?
   1. One time injection
   2. Repeat injection every 1-2 weeks for 3 treatments
   3. Repeat injection based on short-term clinical response (i.e., re-injection performed within 3 months of initial therapy)
   4. Repeat injection based on long-term clinical response (i.e., ‘maintenance’ therapy performed every 6 mo - 1 yr)
   5. Other - Please specify _____________
5. In your population of dogs receiving autologous conditioned serum (IRAP) intra-articularly, how many cases seem to respond?
   1. Few
   2. Some
   3. A lot
   4. All of them
6. In those positive responders, how much clinical improvement do you tend to see?
   1. A little
   2. Some
   3. Substantial
   4. Total resolution
7. When administering autologous conditioned serum (IRAP) intra-articularly, what incidence of acute joint flares have you encountered post-administration? A joint flare is defined as a period of increased disease activity or worsening of clinical signs in a particular joint (e.g., increased joint pain, inflammation, and lameness).
   1. None
   2. 1 in 50 dogs (2%)
   3. 1 in 20 dogs (5%)
   4. 1 in 10 dogs (10%)
   5. 1 in 5 dogs (20%)
   6. 1 in 2 dogs (50%)
   7. All cases

**Autologous Protein Solution (APS – Pro-Stride):**

Autologous protein solution (i.e., Pro-Stride™ APS; nSTRIDE***®*** APS) is an autologous product obtained from the dog’s blood. The blood is first processed using a separator device and centrifugation to obtain plasma with concentrated platelets. The plasma is then harvested and processed in a concentration device that allows exposure of the cellular components of the plasma to polyacrylamide beads enhancing their production of anti-inflammatory proteins during a second centrifugation cycle.

1. Do you use autologous protein solution (Pro-Stride™; nSTRIDE®) to treat your patients? If yes, more questions will follow.
   1. Yes
   2. No

*****If no, the survey jumps to the next topic area**.***

*****If yes, the survey continues on**.***

1. Please rank at least the top 2 most common reasons you use autologous protein solution (Pro-Stride™; nSTRIDE®) in your patients. Click bubbles in order from most important (1) to least (2-7) important reasons.
   1. Preventative or prophylactic measure
   2. Acute articular pathology
   3. Chronic articular pathology needing ‘maintenance’ or routine injections
   4. Post-operative therapy
   5. Ligament or tendon lesions
   6. Tendon sheath or bursa applications
   7. Other - Please specify _____________
2. When using autologous protein solution (Pro-Stride™; nSTRIDE®) intra-articularly, what other additional intra-articular products do you administer simultaneously? If you use more than two products simultaneously, please list the details in the ‘other’ selection.
   1. None
   2. Antibiotic - Please specify product name and dose (mg/kg) _____________
   3. Platelet-rich plasma (PRP) or autologous conditioned plasma (ACP)
   4. Autologous conditioned serum (IRAP)
   5. Cellular therapeutics (stem/stromal/progenitor cell therapy cultured or concentrated tissue particles)
   6. Corticosteroid - Please specify product name and dose (mg/kg) ___________
   7. Viscosupplement (hyaluronic acid, polyacrylamide hydrogel, collagen-elastin, glucosamine, chondroitin)
   8. Radionuclide therapy (Synovetin OA®)
   9. Other - Please specify _____________
3. Do you administer oral or injectable systemic non-steroidal anti-inflammatory medication when administering autologous protein solution (Pro-Stride ™; nSTRIDE®)?
   1. Yes
   2. No
4. Do you ensure that the dog is **not** on any specific medications prior to pulling and processing the autologous protein solution (Pro-Stride ™; nSTRIDE®)?
   1. No
   2. Yes – Please specify which drugs _____________
5. If you use autologous protein solution (Pro-Stride™; nSTRIDE®) intra-articularly, what would your typical treatment protocol be?
   1. One time injection
   2. Repeat injection every 1-2 weeks for 3 treatments
   3. Repeat injection based on short-term clinical response (i.e., re-injection performed within 3 months of initial therapy)
   4. Repeat injection based on long-term clinical response (i.e., ‘maintenance’ therapy performed every 6 mo - 1 yr)
   5. Other - Please specify _____________
6. In your population of dogs receiving autologous protein solution (Pro-Stride™; nSTRIDE®) intra-articularly, how many cases seem to respond?
   1. Few
   2. Some
   3. A lot
   4. All of them
7. In those positive responders, how much clinical improvement do you tend to see?
   1. A little
   2. Some
   3. Substantial
   4. Total resolution
8. When administering autologous protein solution (Pro-Stride™; nSTRIDE®) intra-articularly, what incidence of acute joint flares have you encountered post-administration? A joint flare is defined as a period of increased disease activity or worsening of clinical signs in a particular joint (e.g., increased joint pain, inflammation, and lameness).
   1. None
   2. 1 in 50 dogs (2%)
   3. 1 in 20 dogs (5%)
   4. 1 in 10 dogs (10%)
   5. 1 in 5 dogs (20%)
   6. 1 in 2 dogs (50%)
   7. All cases

**Cellular Therapeutics**

Cellular therapeutics would include the following products:

- Cells (stem/stromal and/or progenitor) contained within tissue particles. These products are typically shipped directly from the company.
- Progenitor and stem/stromal cell concentrates. These products are obtained after harvesting tissue (adipose or bone marrow) and concentration of the cells from the tissue via centrifugation with or without prior tissue digestion (i.e., adipose derived stromal vascular fraction or bone marrow aspirate concentrate).
- Cultured cellular therapy. These products are obtained after harvesting tissue (adipose, bone marrow, blood, etc.) and sending the tissues to a commercial laboratory for culture. The cultured cells would then be shipped back to the practitioner for injection at least 2 weeks or more after the tissue harvest.

1. Do you use cellular therapeutics to treat your patients? If yes, more questions will follow.
   1. Yes
   2. No

*****If no, the survey jumps to the next topic area**.***

*****If yes, the survey continues on**.***

1. Please rank, if possible, at least the top 2 most common reasons you use cellular therapeutics in your patients. Click bubbles in order from most important (1) to least (2-7) important reasons.
   1. Preventative or prophylactic measure
   2. Acute articular pathology
   3. Chronic articular pathology needing ‘maintenance’ or routine injections
   4. Post-operative therapy
   5. Ligament or tendon lesions
   6. Tendon sheath or bursa applications
   7. Other - Please specify _____________
2. When using cellular therapy, what is the most common tissue source that you harvest?
   1. Bone marrow
   2. Adipose – Please specify harvest site _____________
   3. Peripheral blood
   4. Synovial tissues
   5. Other - Please specify _____________
3. When using cellular therapy, what is the donor source for the cellular therapeutic that you are most commonly using?
   1. Autologous (obtained from the same dog that the product is to be used in)
   2. Allogenic (obtained from a different dog than the product is to be used in)
   3. Xenogenic (obtained from a different species)
4. If you use a commercialized system, company or supplier to process or provide cellular therapeutics, which do you most commonly use?
   1. Pure BMC ®, Companion Animal Health
   2. AniCell Biotech
   3. VetStem
   4. Acti-stem Therapy, Ardent Animal Health
   5. Advanced Regenerative Therapies
   6. DogStem ®
   7. Other - Please specify _____________
5. When using cellular therapy intra-articularly, what other additional intra-articular products do you administer simultaneously? If you use more than two products simultaneously, please list the details in the ‘other’ selection.
   1. None
   2. Antibiotic - Please specify product name and dose (mg/kg) ________
   3. Platelet-rich plasma (PRP) or autologous conditioned plasma (ACP)
   4. Autologous conditioned serum (IRAP)
   5. Autologous protein solution (i.e. Pro-Stride ™)
   6. Corticosteroids - Please specify product name and dose (mg/kg) ________
   7. Viscosupplement (hyaluronic acid, polyacrylamide hydrogel, collagen-elastin, glucosamine, chondroitin)
   8. Radionuclide therapy (Synovetin OA®)
   9. Other - Please specify _____________
6. If you use cellular therapy intra-articularly, what would your typical treatment protocol be?
   1. One time injection
   2. Repeat injection every 1-2 weeks for 3 treatments
   3. Repeat injection based on short-term clinical response (i.e., re-injection performed within 3 months of initial therapy)
   4. Repeat injection based on long-term clinical response (i.e., ‘maintenance’ therapy performed every 6 mo - 1 yr)
   5. Other - Please specify _____________
7. In your population of dogs receiving cellular therapy intra-articularly, how many cases seem to respond?
   1. Few
   2. Some
   3. A lot
   4. All of them
8. In those positive responders, how much clinical improvement do you tend to see?
   1. A little
   2. Some
   3. Substantial
   4. Total resolution
9. When administering cellular therapy intra-articularly, what incidence of acute joint flares have you encountered post-administration? A joint flare is defined as a period of increased disease activity or worsening of clinical signs in a particular joint (e.g., increased joint pain, inflammation, and lameness).
   1. None
   2. 1 in 50 dogs (2%)
   3. 1 in 20 dogs (5%)
   4. 1 in 10 dogs (10%)
   5. 1 in 5 dogs (20%)
   6. 1 in 2 dogs (50%)
   7. All cases

**Viscosupplements**

**Hyaluronan**, also referred to as **hyaluronic acid (HA)**, is a naturally occurring component of cartilage and joint fluid that provides lubrication. Intra-articular injection of synthetic HA is believed to be beneficial in decreasing inflammation, repairing cartilage and decreasing symptoms of osteoarthritis (OA). **Polyacrylamide hydrogel** is a synthetic viscosupplement product injected intra-articularly. It is incorporated into the synovial lining and provides enhanced viscoelasticity to the synovial fluid. **Collagen-elastin** products are injected with the aim of providing a scaffold for intra-articular space. Other viscosupplements for intra-articular injection include **glucosamine** and **chondroitin**. Viscosupplements products for intra-articular injection include the following among others:

- Arthramid ® Vet, Contura Vet
- Hyalovet ®, Boehringer Ingelheim
- Hylartin-V ®, Zoetis
- Hyvisc®, Boehringer Ingelheim
- Legend®, Boehringer Ingelheim
- Noltrex®Vet, Nucleus ProVets
- Polyglycan®, SA, Bimeda
- Synamid®, Contura Vet
- Synvisc® (Hylan G-F 20), Sanofi
- Spryng^TM^, PetVivo Holdings, Inc

1. Do you use viscosupplements to treat your patients? If yes, more questions will follow.
   1. Yes
   2. No

*****If no, the survey jumps to the next topic area**.***

*****If yes, the survey continues on**.***

1. Which viscosupplement product(s) do you most commonly use? Select all that apply.
   1. Arthramid® Vet, Contura Vet
   2. Hyalovet®, Boehringer Ingelheim
   3. Hylartin-V®, Zoetis
   4. Hyvisc®, Boehringer Ingelheim
   5. Legend®, Boehringer Ingelheim
   6. Noltrex®Vet, Nucleus ProVets
   7. Polyglycan®, SA, Bimeda
   8. Synamid®, Contura Vet
   9. Synvisc® (Hylan G-F 20), Sanofi
   10. Spryng^TM^, PetVivo Holdings, Inc
   11. Other - Please specify _____________
2. Please rank at least the top 2 most common reasons you use viscosupplements products in your patients. Click bubbles in order from most important (1) to least (2-5) important reasons.
   1. Preventative or prophylactic measure
   2. Acute articular pathology
   3. Chronic articular pathology needing ‘maintenance’ or routine injections
   4. Post-operative therapy
   5. Other - Please specify _____________
3. When using a viscosupplement intra-articularly, what other additional intra-articular products do you administer simultaneously? If you use more than two products simultaneously, please list the details in the ‘other’ selection.
   1. None
   2. Antibiotics - Please specify product name and dose (mg/kg) ________
   3. Platelet-rich plasma (PRP) or autologous conditioned plasma (ACP)
   4. Autologous conditioned serum (IRAP)
   5. Autologous protein solution (i.e., Pro-Stride ™)
   6. Cellular therapeutics (stem/stromal/progenitor cell therapy cultured or concentrated tissue particles)
   7. Corticosteroids - Please specify product name and dose (mg/kg) ________
   8. Other viscosupplement - Please specify product name ________
   9. Radionuclide therapy (Synovetin OA®)
   10. Other - Please specify _____________
4. If you use a viscosupplement intra-articularly, what would your typical treatment protocol be?
   1. One time injection
   2. Repeat injection every 1-2 weeks for 3 treatments
   3. Repeat injection based on short-term clinical response (i.e., re-injection performed within 3 months of initial therapy)
   4. Repeat injection based on long-term clinical response (i.e., ‘maintenance’ therapy performed every 6 mo - 1 yr)
   5. Other - Please specify _____________
5. In your population of dogs receiving viscosupplements intra-articularly, how many cases seem to respond?
   1. Few
   2. Some
   3. A lot
   4. All of them
6. In those positive responders, how much clinical improvement do you tend to see?
   1. A little
   2. Some
   3. Substantial
   4. Total resolution
7. When administering viscosupplements intra-articularly, what incidence of acute joint flares have you encountered post-administration? A joint flare is defined as a period of increased disease activity or worsening of clinical signs in a particular joint (e.g., increased joint pain, inflammation, and lameness).
   1. None
   2. 1 in 50 dogs (2%)
   3. 1 in 20 dogs (5%)
   4. 1 in 10 dogs (10%)
   5. 1 in 5 dogs (20%)
   6. 1 in 2 dogs (50%)
   7. All cases

**Radionuclide**

Radiosynoviorthesis (RSO) is a technique used to restore the joint fluid by intra-articular injection of radioactive agents. Tin-117m colloid in the product Synovetin OA^®^ is the most novel RSO device used to treat synovial inflammation, improve mobility and mitigate osteoarthritis in dogs.

1. Do you use radiosynoviorthesis (RSO) [Synovetin OA^®^] therapy to treat your patients? If yes, more questions will follow.
   1. Yes
   2. No

*****If no, the survey jumps to the end**.***

*****If yes, the survey continues on**.***

1. Please rank at least the top 1 most common reason you use radiosynoviorthesis (RSO) in your patients. Click bubbles in order from most important (1) to least (2-5) important reasons.
   1. Preventative or prophylactic measure
   2. Acute articular pathology
   3. Chronic articular pathology needing ‘maintenance’ or routine injections
   4. Post-operative therapy
   5. Other - Please specify _____________
2. When using radionuclide therapy (Synovetin OA^®^) intra-articularly, what other additional intra-articular products do you administer simultaneously? If you use more than two products simultaneously, please list the details in the ‘other’ selection.
   1. None
   2. Antibiotic - Please specify product name and dose (mg/kg) ________
   3. Platelet-rich plasma (PRP) or autologous conditioned plasma (ACP)
   4. Autologous conditioned serum (IRAP)
   5. Autologous protein solution (i.e., Pro-Stride ™)
   6. Cellular therapeutics (stem/stromal/progenitor cell therapy cultured or concentrated tissue particles)
   7. Corticosteroid - Please specify product name and dose (mg/kg) ________
   8. Viscosupplement (hyaluronic acid, polyacrylamide hydrogel, collagen-elastin, glucosamine, chondroitin)
   9. Other - Please specify _____________
3. If you use radionuclide therapy (Synovetin OA^®^) intra-articularly, what would your typical treatment protocol be?
   1. Once per 12-month interval in elbow joints only
   2. Once per 12-month interval in joints other than the elbow
   3. Other - Please specify _____________
4. In your population of dogs receiving radionuclide therapy (Synovetin OA^®^) intra-articularly, how many cases seem to respond?
   1. Few
   2. Some
   3. A lot
   4. All of them
5. In those positive responders, how much clinical improvement do you tend to see?
   1. A little
   2. Some
   3. Substantial
   4. Total resolution
6. When administering radionuclide therapy (Synovetin OA^®^) intra-articularly, what incidence of acute joint flares have you encountered post-administration? A joint flare is defined as a period of increased disease activity or worsening of clinical signs in a particular joint (e.g., increased joint pain, inflammation, and lameness).
   1. None
   2. 1 in 50 dogs (2%)
   3. 1 in 20 dogs (5%)
   4. 1 in 10 dogs (10%)
   5. 1 in 5 dogs (20%)
   6. 1 in 2 dogs (50%)
   7. All cases

**Other non-steroidal intra-articular therapeutics**

1. Would you like to report any other non-steroidal intra-articular therapeutic(s) not mentioned in our survey that you as a practitioner use routinely and find helpful in the management of canine osteoarthritis?
   1. No
   2. Yes - Please specify which one(s) _____________

We thank you and appreciate your time spent taking this survey. Your response has been recorded. Please feel free to close the window at your convenience.
